# The comparative transient prediction on hydrodynamic characteristics and flow field properties of pump-jets with accelerating and decelerating ducts

**DOI:** 10.1038/s41598-024-54388-z

**Published:** 2024-03-02

**Authors:** Yunkai Zhou, Jianping Yuan, Giovanna Cavazzini, Yanxia Fu, Quanlin Gao

**Affiliations:** 1 National Research Center of Pumps, Jiangsu University, Zhenjiang, 212013 China; 2 Department of Industrial Engineering, University of Padua, Via Venezia 1, 35131 Padua, Italy; 3 Institute of Fluid Engineering Equipment, JITRI, Jiangsu University, Zhenjiang, 212013 China; 4 School of Energy and Power Engineering, Jiangsu University, Zhenjiang, 212013 China

**Keywords:** Pump-jet, Hydrodynamic performance, Transient analysis, Accelerating duct, Decelerating duct, Engineering, Mechanical engineering

## Abstract

Pump-jet holds a pivotal position in various marine applications, underscoring the need for comprehending their transient behavior for the purpose of design enhancement and performance refinement. This paper employs Reynolds-averaged Navier–Stokes equations method in conjunction with Detached Eddy Simulation model. The study delves into the ramifications of accelerating and decelerating ducts, distinguished by camber *f* and attack angles *α*, on transient hydrodynamic characteristics. The hydrodynamic characteristics are investigated numerically, after the validation of the numerical methodology by comparing simulation outcomes against experimental results. Subsequently, the study delves into propulsion characteristics, followed by an exploration of time-domain and frequency-domain data transformed through fast Fourier transform to analyze thrust fluctuations and pulsating pressures. Additionally, a detailed examination of pressure distribution and velocity field is provided, aiming to dissect the mechanisms through the variations in *f* and *α* influence the flow field. Findings suggest that the outlet velocity of accelerating ducts significantly surpasses the inlet velocity, a behavior contrasted by decelerating ducts. Notably, the patterns of accelerating and decelerating ducts resulting from alterations in *f* exhibit consistent characteristics with those brought about by changes in *α*. However, several opposite characteristics surface in transient flow field due to the distinct modifications in the duct profile. Furthermore, by considering vorticity magnitude distribution and vortices, a comparative analysis elucidates the effects of varying *f* and *α* on rotor and stator trailing vortices. This contributes to understanding the flow instability mechanism under differing duct configurations. It is evident that changes in *f* and *α* exert significant influence on both performance and flow field.

## Introduction

The pump-jet, encompassing a rotor, stator, and duct, represents a highly promising alternative propulsor equipped on underwater vehicles. Its notable superiorities, when compared to other propulsor methods, include elevated critical speed, superior propulsion efficiency, robust anti-cavitation performance, and minimal radiated noise under operational conditions. The duct structural configuration serves a variety of critical functions, such as safeguarding internal blades, providing acoustic shielding and enhancing hydrodynamic efficiency in the case of an accelerating duct, or mitigating cavitation generation in the case of a decelerating duct. Nevertheless, the complexity of the pump-jet multiple components and the non-uniform wake flow engender intricate hydrodynamic interactions. These interactions can give rise to adverse consequences, including cavitation erosion, the instigation of pressure fluctuations, structural vibrations, and the emission of underwater noise.

The initial exploration into pump-jet design and performance was undertaken in 1963 by McCormick et al.^1^, marking the inception of duct determination. Additionally, the inaugural public treatment of numerical analysis pertaining to pump-jet utilization in torpedoes was presented by Ivanell^2^. The inclusion of a duct within a pump-jet structure segregates the fluid flow into two distinct categories, including the inner and outer fluid. The duct profile section induces axial component forces owing to the pressure difference between the duct inner and outer surfaces^3^. A thorough investigation involving the selection and influence of critical duct parameters can be effectively carried out using both numerical and experimental approaches. The analysis of these pivotal parameters is significant as they yield diverse effects on the pump-jet and contribute to intricate interaction dynamics. In recent studies, Huang et al.^4^ and Wang et al.^5^ delved into the hydrodynamic performance of pump-jets, considering various duct parameters. The outcomes gleaned from these investigations underscore the significant and intricate impact on pump-jet hydrodynamic performance stemming from variations in duct parameters such as camber, tip-clearance size, duct attack angle, length-diameter ratio, and the expansion ratio of the pump-jet outlet.

Regarding the classification of ducts, the manipulation of airfoil shape or inlet angle facilitates the regulation of the duct to generate resistance, thrust, or neutral force, leading to the categorization of ducts as either accelerating or decelerating effects^6–9^. Meanwhile, Distinct structural characteristics differentiate accelerating and decelerating ducts, chiefly attributed to variations in cambers and angles of attack. In the context of accelerating ducts, the internal inlet diameter exceeds that of the outlet, whereas for decelerating ducts, the inlet internal diameter is smaller than the outlet. Broadly, the accelerating duct finds application in scenarios of high propulsion load and low speed. It generates substantial positive thrust, thereby fostering enhanced propulsion efficiency. However, this advantage comes at the cost of diminished anti-cavitation capability due to low pressures at the duct’s internal surface^8^. In contrast, the decelerating duct serves the purpose of retarding cavitation and mitigating associated negative impacts such as noise and vibrations^7^. However, the decelerating duct augments drag, necessitating higher blade thrust and leading to reduced hydrodynamic efficiency. Limited literature delves into performance studies of both accelerating and decelerating ducts, with comparative research being particularly scarce. Gaggero et al.^10,11^ highlight the effects of duct configuration on propulsion, noting that accelerating ducts primarily boost propeller efficiency, particularly in heavily loaded conditions. In contrast, decelerating ducts mitigate cavitation erosion, enhancing noise performance and suppressing vibrations. Notably, Gaggero et al. employ B-Spline-derived duct profiles subjected to shape manipulation through control points. They utilize axial momentum theory and a nonlinear semi-analytical actuator disk model to investigate duct parameters like length, camber, and maximum thickness^7,8,12^. While the investigations mentioned primarily focus on ducts used with ducted propellers and the corresponding flow fields, limited attention has been directed towards evaluating the impacts of accelerating and decelerating ducts on pump-jet performance and overall interactions within the pump-jet system.

The prediction of pump-jet performance and flow characteristics hinges on two primary methodologies: numerical simulations and experimental studies. Numerical simulations offer advantages such as cost-efficiency and clearer visualization of flow properties, rendering them more prolific and sophisticated than experimental approaches. Suryanarayana et al.^13,14^ undertake both numerical and experimental investigations of a pump-jet propulsor for an axisymmetric body within a wind tunnel. Their findings demonstrate that Computational Fluid Dynamics (CFD) provides an economical and reasonably accurate means for swiftly predicting overall pump-jet performance. While these early studies lay foundational insights into pump-jet research, the intricate composition of pump-jet components and the non-uniform wake flow in practical scenarios lead to adverse effects such as cavitation, fluctuating pressures, vibrations, and underwater radiated noise. Consequently, researchers have increasingly delved into in-depth explorations of pump-jet hydrodynamic characteristics and flow fields in recent times. To accurately simulate flow dynamics within the pump-jet propulsor, Li et al.^15^ focus on unsteady hydrodynamics performance, transient flow fields, and vortex wake comparison by employing the RANS (Reynolds-Averaged Navier–Stokes) and hybrid RANS/LES (Large Eddy Simulation) categories. Their conclusions highlight that the Detached Eddy Simulation (DES) method captures more comprehensive turbulence structures and better replicates turbulent properties compared to the RANS solver. Additionally, another study by Li et al.^16^ adopts the DES method to explore properties of cavitation and flow fields in pump-jet propulsion. The results underscore that when cavitation occurs, the intensity of Tip Leakage Vortices (TLVs) downstream of non-cavitating regions increases along with the growth of the Tip Sheet Vortices (TSVs), leading to compromised propulsion performance, heightened vibrations, radiated noise, and cavitation erosion. Building upon prior investigations, these studies demonstrate that duct-related analysis contributes to enhanced capabilities in resolving rotor wake vortices.

Presently, the task of devising ducts for pump-jet systems and harmonizing their integration with the overall propulsion structure remains a complex endeavor. This challenge is compounded by the presence of unstable flow dynamics and intricate interactions between the duct and adjacent components. This exploration encompasses comprehending the impacts introduced by accelerating and decelerating ducts on the instantaneous characteristics within the pump-jet flow environment, as well as unraveling the mechanisms driving instabilities in trailing vortices.

In order to address the aforementioned challenge, this study endeavors to explore the instantaneous effects of distinct accelerating and decelerating ducts characterized by cambers *f* and angles of attack *α*. In “Design and modeling” section, to discern the distinct contributions of various duct designs, a set of models featuring both accelerating and decelerating ducts is established. These models are systematically modified using five different values of *f* and three different* α*. In “Numerical methodology” section, a combination of the Reynolds-averaged Navier–Stokes equations (RANSE) method and the Detached Eddy Simulation (DES) model employed in this paper is introduced. To ensure the accuracy and reliability of subsequent numerical simulation, meticulous attention is given to the creation of a precise structural mesh for the entire computational domain. Subsequently, a comprehensive assessment ensues, encompassing a comparative analysis of both experimental and simulated outcomes for non-cavitating scenarios involving propeller VP1304 in “Grid generation and numerical set up” section, as well as the pump-jet operating in mooring conditions. The ensuing analytical discussion, presented in “Results and discussion” section, delves into the intricate nuances of the flow characteristics. It includes a thorough exhibition of the open water performance and transient flow field attributes of the pump-jet configuration, considering both the accelerating and decelerating duct variations governed by the modified *f* and *α*. Finally, the research culminates in the formulation of conclusions, eloquently outlined in “Conclusion” section.

## Design and modeling

### Design of duct profile

**Figure 1:**
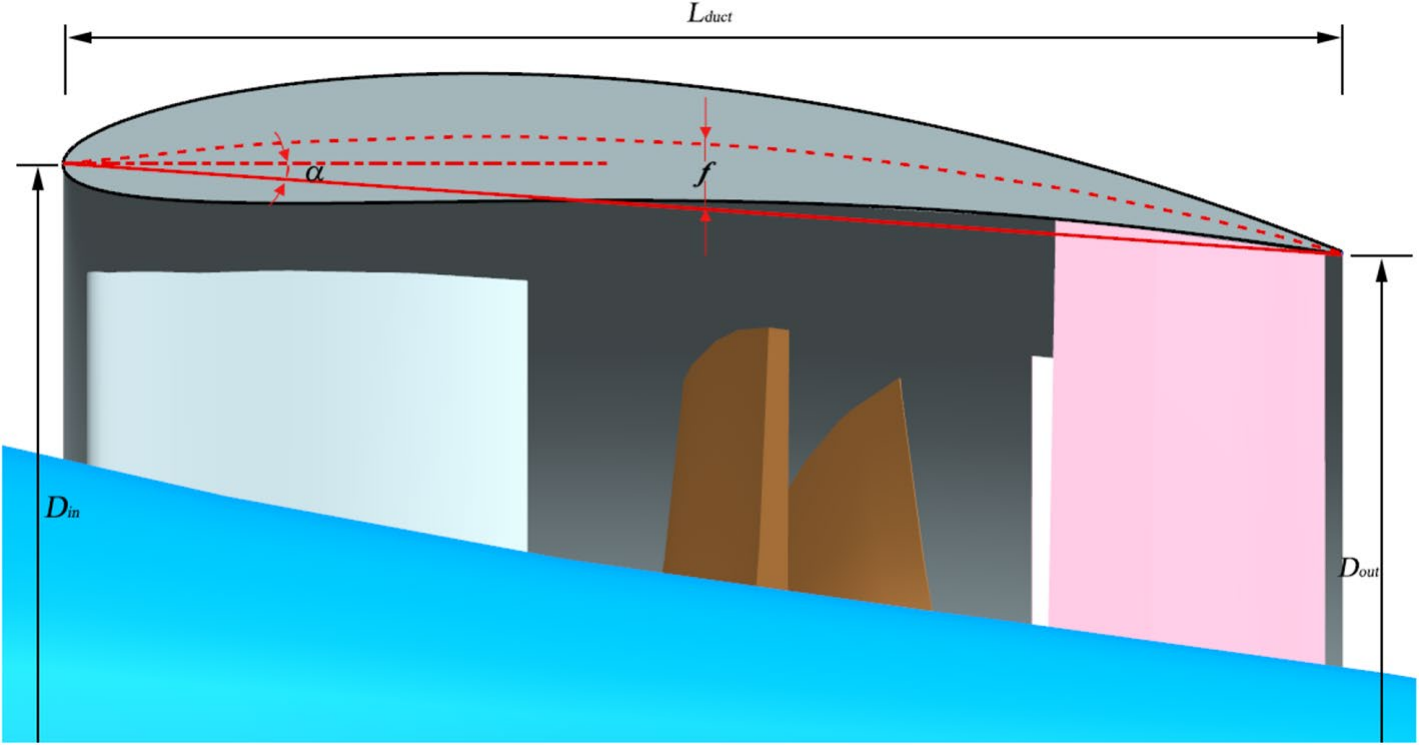
The diagrammatic sketch of the parameters which distinguish accelerating and decelerating ducts.

**Figure 2:**
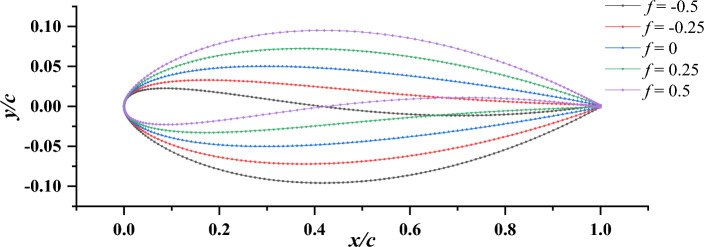
Schematic diagram of duct profile with different cambers.

**Figure 3:**
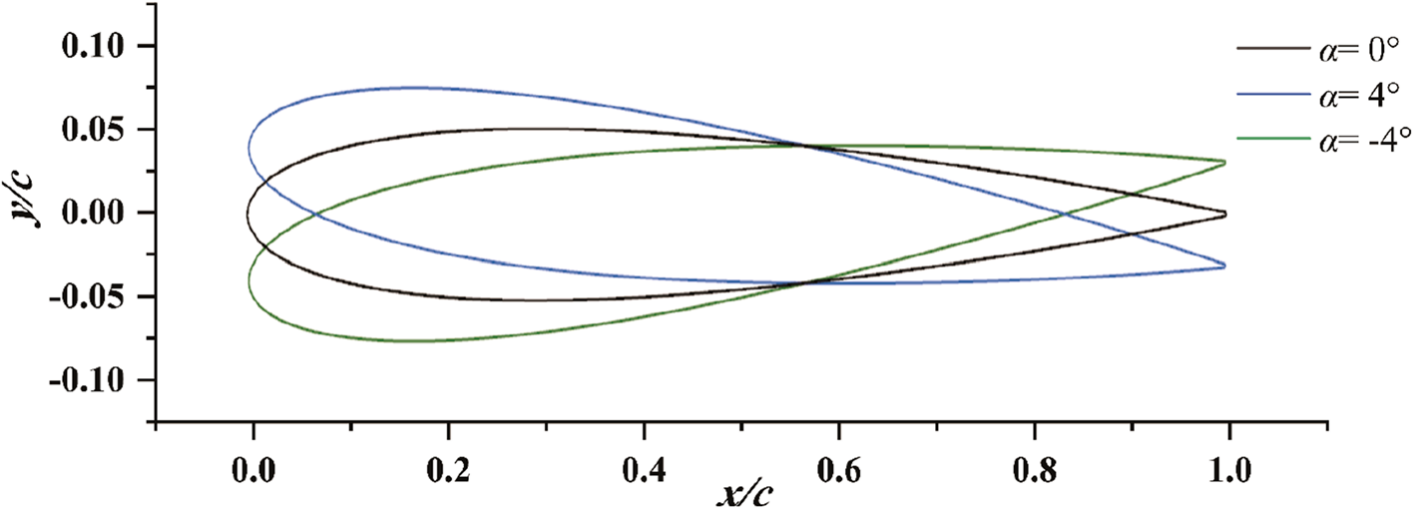
Schematic diagram of duct profile with different angle of attack.

Due to the presence of the duct, the flow is split into the inner flow field and outer flow field. Meanwhile, the flow field undergoes changes due to variations in the duct profile configuration. In order to differentiate between accelerating and decelerating ducts and establish their structural disparities, the characterization can be attributed to two key aspects: variations in camber and angles of attack^6,17^. Moreover, a prominent structural difference between them lies in the fact that the accelerating duct exhibits a larger area at the inlet compared to the outlet, while the decelerating duct showcases the opposite structural configuration. Thus, in this investigation, the variables *f* and *α* are adopted in the duct profile design of the pump-jet, as indicated in Fig. 1. Due to the non-parallel orientation of the hull and inner hub of the pump-jet with the horizontal plane, the hull surface is chosen as the reference horizontal surface for adjusting parameters *f* or *α* during the investigation of the effects of accelerating and decelerating ducts. Consequently, when categorizing the ducts based on variations in camber and their alignment with the pump-jet hub, the airfoil sections of the decelerating and accelerating ducts are set at chord angles of 4° and 11.5°, respectively. The angle of attack is then adjusted from the initial chord angle of 11.5°^18^. To comprehensively assess the impact of accelerating and decelerating ducts on pump-jet performance, different configurations of the physical model have been investigated. Various camber sizes (*f* = 0.5t, 0.25t, 0, − 0.25t, − 0.5t) and angle of attack values (*α* = 4°, 0°, − 4°) are chosen. Figure 2 illustrates the variation and comparison of the initial duct profile camber at the same level. However, to meet specific requirements, the duct profile will rotate around the leading edge’s vertex. Additionally, for altering the angle of attack, the duct profile rotates around the midpoint in the axial direction of the impeller, as shown in Fig. 3. It depicts the schematic of the duct profile with varying angles of attack at the same level. It is important to note that the tip clearance size remains fixed at 3 mm, controlled by securing the internal components of the pump-jet and adjusting the duct profile’s position along the y-axis direction. This ensures that the outcomes remain unaffected by other factors.

### Physical model of pump-jet

**Figure 4:**
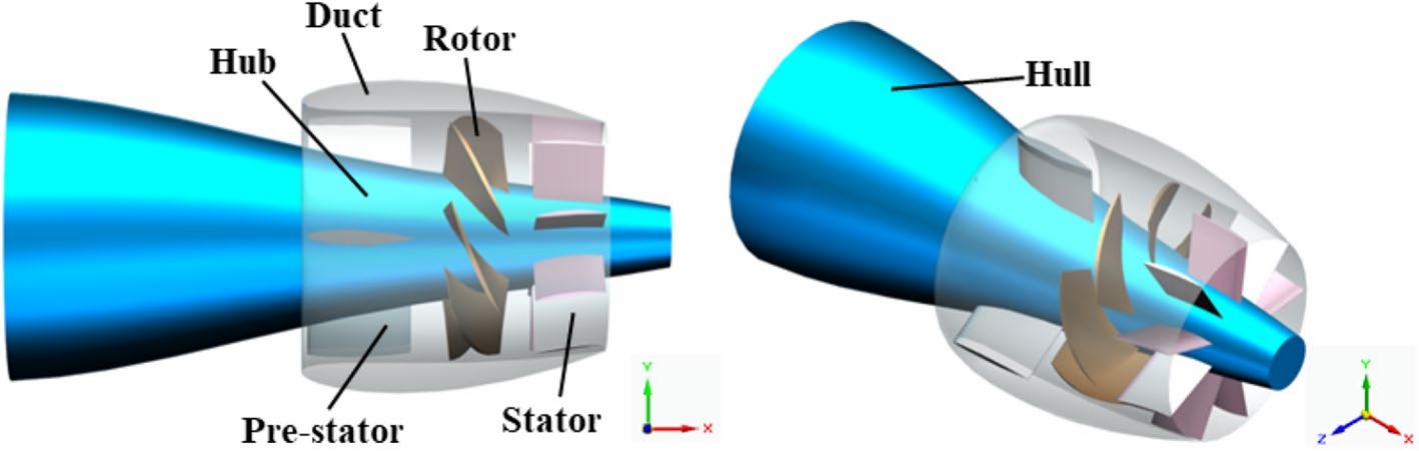
Geometry model of pump-jet propulsion.

After modeling the duct, nine stator blades are securely connected to the duct and hub. Inside the duct, there are three pre-stator blades and seven rotor blades, arranged in the sequence of pre-stator, rotor, and stator. In this study, the computational geometry configuration of the prototype pump-jet is depicted in Fig. 4. In this study, the pre-stator with 3 blades serves a dual purpose, functioning not only as a pre-swirl guide but also as a support frame. Furthermore, the rotation of rotor occurs around the x-axis at a speed of 2400 r/min, aligned with the x-axis direction. The y-axis signifies the vertically upward direction, which is crucial for adjusting the tip clearance. In order to derive more universally applicable conclusions, this research employs the NACA5510 airfoil as a simplified duct profile for the pump-jet, and specific parameters were selected with reference to reference^17^. The initial shape value corresponds to a profile with a camber of *f* = 0.5t, with ‘*t*’ signifying the airfoil thickness^18^. Additionally, for the purpose of simplifying the presentation of the fundamental elements and constituents of the pump jet propulsion system, the geometrical representations displayed in Figs. 1 and 4 portray the pump-jet equipped with the initial duct profile configuration—a pump-jet featuring a duct profile of *f* = 0.5t and *α* = 0°.

In this investigation, a dimensionless methodology is employed to evaluate and compare the hydrodynamic characteristics of the pump-jet propulsion system. This approach involves utilizing relevant physical performance parameters in a dimensionless manner. The outcomes of the numerical simulations are presented in the previous research, illustrated in Table 1, which enables a clear representation of the results^17^. In the table, it illustrates the allocation of components within the system. The dynamic elements, designated by the subscript ‘*r*,’ encompass the rotor, while the pre-stator, stator, and duct constitute the stationary components indicated by the subscript ‘*s*’. The performance metrics are represented by *T* for thrust and *Q* for torque. It is evident from the equation provided that adjusting the advance coefficient *J* can be achieved by manipulating both the inflow speed *U* and the rotor rotational speed *n*.

**Table 1:** Dimensionless presentation of pertinent physical performance parameters.

Physical parameter	Definition
Advance coefficient	$J = U/\left( {nD} \right)$
Thrust coefficient of rotating system	$K_{{T_{r} }} = T_{r} /\left( {\rho n^{2} D^{4} } \right)$
Thrust coefficient of static system	$K_{{T_{s} }} = T_{s} /\left( {\rho n^{2} D^{4} } \right)$
Torque coefficient of rotating system	$K_{{Q_{r} }} = Q_{r} /\left( {\rho n^{2} D^{5} } \right)$
Total thrust coefficient	$K_{T} = K_{{T_{r} }} + K_{{T_{s} }}$
Total torque coefficient	$K_{Q} = K_{{Q_{r} }}$
Propulsion efficiency	$\eta = \left( {J \cdot K_{T} } \right)/2\pi \cdot K_{Q}$

The pressure coefficient, denoted as *Cp*, is employed as a dimensionless parameter to characterize the distribution of pressure within the system.

1 $$\begin{array}{*{20}c} {C_{p} = \frac{{P - P_{\infty } }}{{0.5\rho_{l} \left( {\pi nD} \right)^{2} }}} \\ \end{array}$$

where the fluid density is denoted as *ρ*_l_, and the symbol *p*_∞_ represents the far-field pressure, which is taken as the outlet pressure value within the calculation domain for the purposes of this study.

## Numerical methodology

### Governing equations

In this study, the fluid is treated as a single-phase, incompressible medium under the assumption of homogeneity. The time-averaged continuity equation and momentum conservation equation, excluding body forces and gravitational acceleration, can be expressed as follows

2 $$\begin{array}{*{20}c} {\frac{\partial \rho }{{\partial t}} + \frac{{\partial (\rho u_{i} )}}{{\partial x_{i} }} = 0} \\ \end{array}$$

3 $$\begin{array}{*{20}c} {\frac{\partial }{\partial t}\left( {\rho u_{i} } \right) + \frac{{\partial (\rho u_{i} u_{j} )}}{{\partial x_{j} }} = {-}\frac{\partial p}{{\partial x_{i} }} + \frac{\partial }{{\partial x_{i} }}\left[ {\mu \frac{{\partial u_{i} }}{{\partial x_{j} }} - \rho \overline{{u_{i}^{\prime } u_{j}^{\prime } }} } \right] + S_{i} } \\ \end{array}$$

where *ρ* is the fluid medium density, *μ* denotes the fluid dynamic viscosity, $u_{i}^{\prime } u_{j}^{\prime }$ corresponds to the Reynolds stresses, *p* represents the pressure and *u* signifies the time averaged velocity. Additionally, *x* refers to the spatial coordinate, while *S* denotes the source term. The subscript *i* and *j* indicate the coordinate component.

### Turbulence model

The DES model (Detached Eddy Simulation) is a representative RANS model known for its sensitivity to grid spacing. It is a hybrid approach specifically designed for the analysis of unsteady flow, where it selectively employs URANS or LES in appropriate regions of the computational domain^19^. In areas with insufficient grid resolution, DES resorts to the unsteady RANS model upon which it is based. However, in high Reynolds number regions, particularly those far from solid boundaries, DES operates in the LES mode. Therefore, when addressing the various boundary layers in a pump-jet system, the DES model is a relatively suitable and precise choice for numerical simulations. Consequently, the turbulence model adopted in this study is the DES model based on SST *k*–*ω* to simulate the flow characteristics of the pump-jet. The equations for the k and ω terms in the SST *k*–*ω* model are as follows,

4 $$\begin{array}{*{20}c} {\frac{{\partial \left( {\rho k} \right)}}{\partial t} + \rho u_{i} \frac{\partial k}{{\partial x_{i} }} = P_{k} - \rho \frac{{k^{3/2} }}{{l_{RANS} }} + \frac{\partial }{{\partial x_{j} }}\left[ {\left( {\mu + \frac{{\mu_{t} }}{{\sigma_{k} }}} \right)\frac{\partial k}{{\partial x_{j} }}} \right]} \\ \end{array}$$

5 $$\begin{aligned} & \rho \frac{\partial \omega }{{\partial t}} + \rho u_{i} \frac{\partial \omega }{{\partial x_{i} }} = \frac{\partial }{{\partial x_{j} }}\left[ {\left( {\mu + \frac{{\mu_{t} }}{{\sigma_{k} }}} \right)\frac{\partial \omega }{{\partial x_{j} }}} \right] + C_{\omega } P_{\omega } - \beta_{\omega } \rho \omega^{2} \\ & \quad + \left( {1 - F_{1} } \right)2\rho \frac{1}{{\omega \sigma_{\omega 2} }}\frac{\partial k}{{\partial x_{j} }}\frac{\partial \omega }{{\partial x_{j} }} \\ \end{aligned}$$

where the dimensionless eddy viscosity factor *µ*_*t*_ is

6 $$\begin{array}{*{20}c} {\mu_{t} = min\left[ {\frac{\rho k}{\omega },\frac{{a_{1} \rho k}}{{F_{2} \Omega }}} \right]} \\ \end{array}$$

Otherwise, *F*_1_ represents the weighting function, *F*_2_ represents the mixing function, *P*_ω_ and *P*_k_ are the dissipation source and turbulence generation terms caused by viscous force, while *S* represents the curl amplitude.

The turbulence scale parameter *l*_RANS_ in the dissipation term of the k equation is defined as follow

7 $$\begin{array}{*{20}c} {l_{RANS} = \frac{{k^{1/2} }}{{\beta_{k} \omega }}} \\ \end{array}$$

In the context of the DES method, the turbulence scale parameter *l*_*RANS*_ is substituted with the DES scale parameter, denoted as *l*_*DES*_. The symbol *Δ* represents the maximum grid scale value in all three directions, suitable for a heterogeneous grid. The constant *C*_DES_ is a model coefficient determined through the mixing function, typically assigned a value of 0.65.

8 $$\begin{array}{*{20}c} {l_{DES} = min\left( {l_{RANS} ,l_{LES} } \right)} \\ \end{array}$$

9 $$\begin{array}{*{20}c} {l_{LES} = C_{DES} \Delta } \\ \end{array}$$

10 $$\begin{array}{*{20}c} {\Delta = max\left\{ {\Delta x,\Delta y,\Delta z} \right\}} \\ \end{array}$$

In the near-wall boundary layer, where *l*_*DES*_ equals *l*_*RANS*_ and the dissipation term for the turbulent kinetic energy *k* is the same as for the conventional RANS, namely the DES model switches to SST *k*–*ω* turbulence model. In regions distant from the wall, where *l*_*DES*_ equals *l*_*LES*_ and the dissipation term of the transport equation becomes (*ρk*^3/2^)/(Δ*C*_*DES*_), employing the Subgrid Reynolds Stress Model within the framework of large eddy simulation.

## Grid generation and numerical set up

### Computational domains and boundary conditions

**Figure 5:**
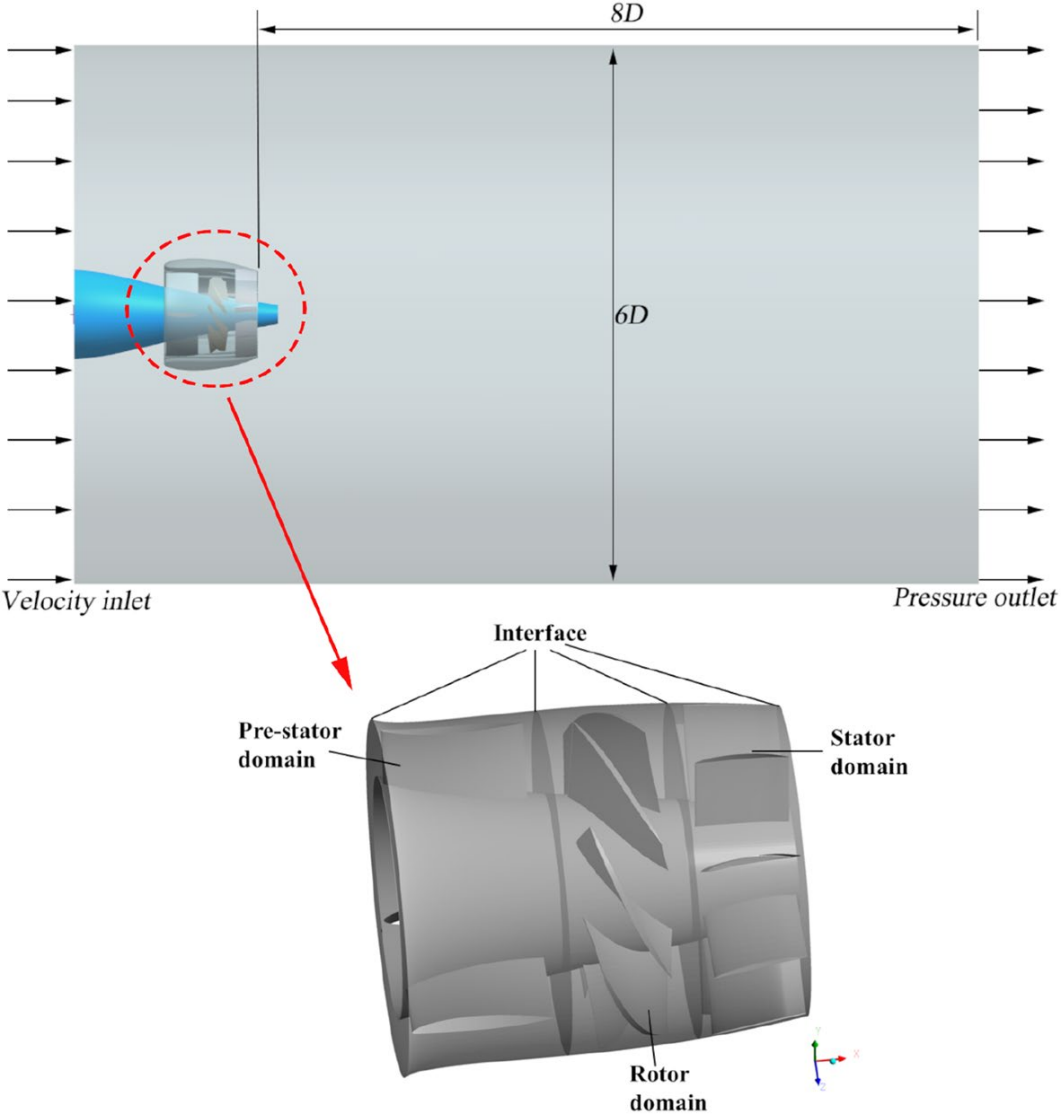
The Computational domain and boundary conditions of pump-jet.

To simulate the underwater operation of the pump-jet, a substantial cylindrical computational domain is designated, enveloping the pump-jet itself. This domain is divided into four distinct areas: the pre-stator domain, rotor domain, stator domain, and external flow field domain. The variable *D* signifies the maximum diameter of the pump-jet rotor. As depicted in Fig. 5, the external flow field outlet is positioned at a distance of 8*D* from the pump-jet outlet, while the inlet is arranged approximately 2*D* upstream of the pump-jet inlet. The overall diameter of the external flow field domain measures 6*D*. While the rotor domain is designed to rotate, the pre-stator domain, stator domain, and external flow field domain remain stationary. These subdomains centerlines coincide due to the horizontal inflow. Furthermore, local grid refinement is implemented within the internal domains and around the duct, enhancing simulation accuracy by addressing intricate flows concentrated in close proximity to the pump-jet.

The configuration of boundary conditions plays a pivotal role in determining the comprehensiveness and accuracy of the numerical simulation. This study employs specific boundary conditions tailored to its requirements. The reference pressure in the computational domain is set to 1 atm. The inlet of the external flow field is designated as a speed inlet with 5% turbulence intensity, while the pump-jet outlet is specified as a static pressure outlet. The cylindrical surface is treated as the free slip wall, whereas non-slip wall conditions are applied to other surfaces such as the duct and blades. Aiming at facilitating interaction and flow field transmission, the frozen rotor model is employed between the pre-stator and rotor domains, as well as between the rotor and stator domains. The solution time is set to 1000 iterations, with a convergence criterion residual target of 10^–5^. Second-order backward euler is chosen as the transient scheme, and the numerical simulation is performed in the discrete format of second-order upwind. The rotational speed of rotor domain is kept constant at *n* = 2400 r/min across all configurations. Additionally, for unsteady numerical simulations, a time step of Δ*t* = 1.3889 × 10^−4^ s is utilized. This corresponds to the rotation of the blade by 2° in a single circle period.

### Monitoring points setting

**Figure 6:**
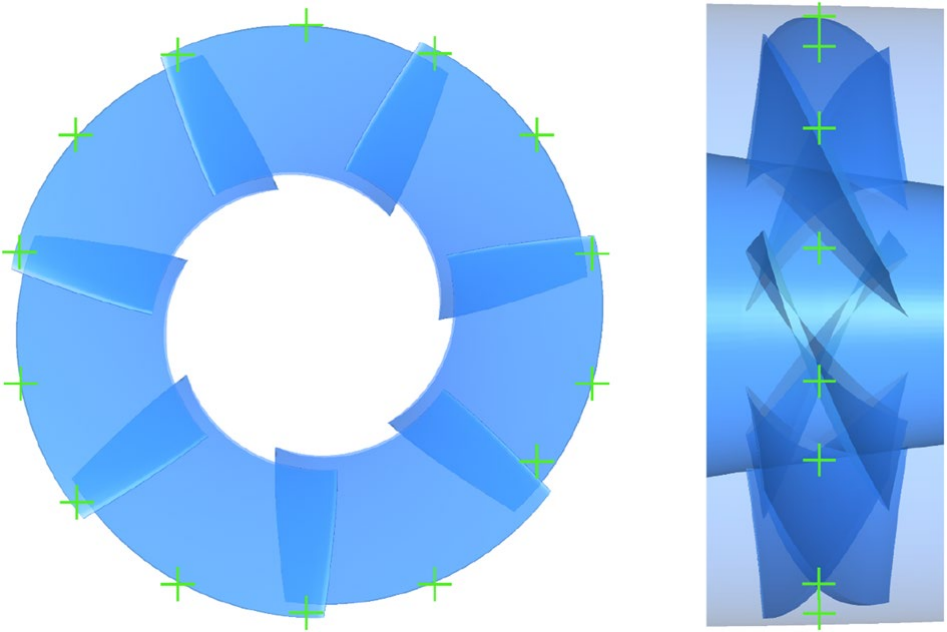
Configuration of monitoring points around the rotor (distributed at the blade tip and blade passage).

Figure 6 indicates the arrangement of monitoring points, which are equally distributed at 360/14° between the duct inside surface and rotor tip. And the monitoring points chosen are composed of two groups. The location of the monitoring points is arranged for the purpose of obtaining the spatial distribution characteristics and development of the pressure fluctuation in the tip clearance flow, which is located in the tip clearance region and the channel between neighboring blades, as well as saving computational resources. Among them, Group 1 (P1, P3, P5, P7, P9, P11, P13) is set at the mid point of rotor blade, named by odd numbers. While located in the passage between blades, there are corresponding points (P2, P4,…P14) named by even numbers, which are Group 2. The points in Group 1 are apart from Group 2 by 360/14 degrees.

### Grid generation and grid independence verification

**Figure 7:**
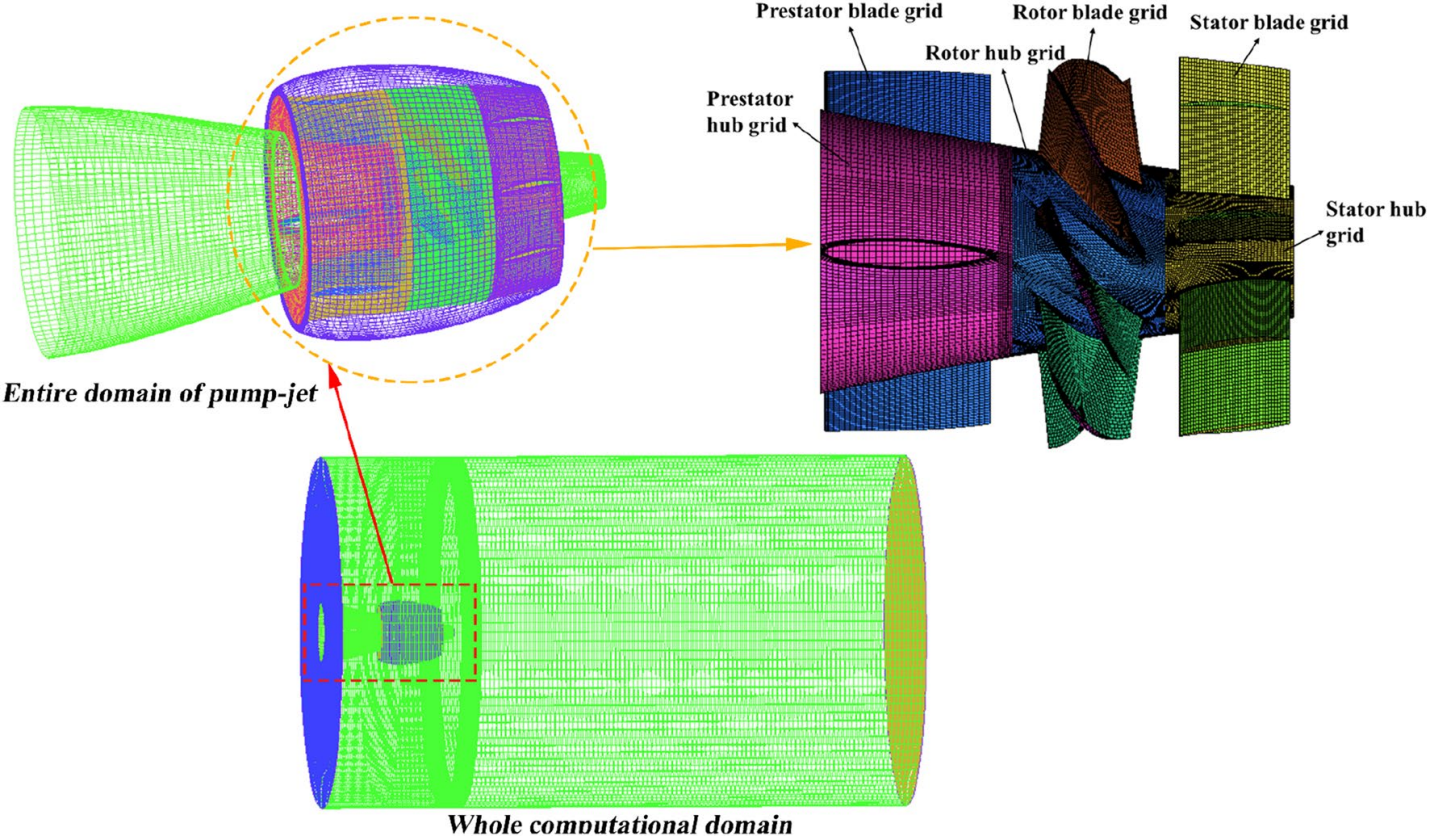
Structured grids of the entire computational domain with the internal domain of pump-jet.

**Figure 8:**
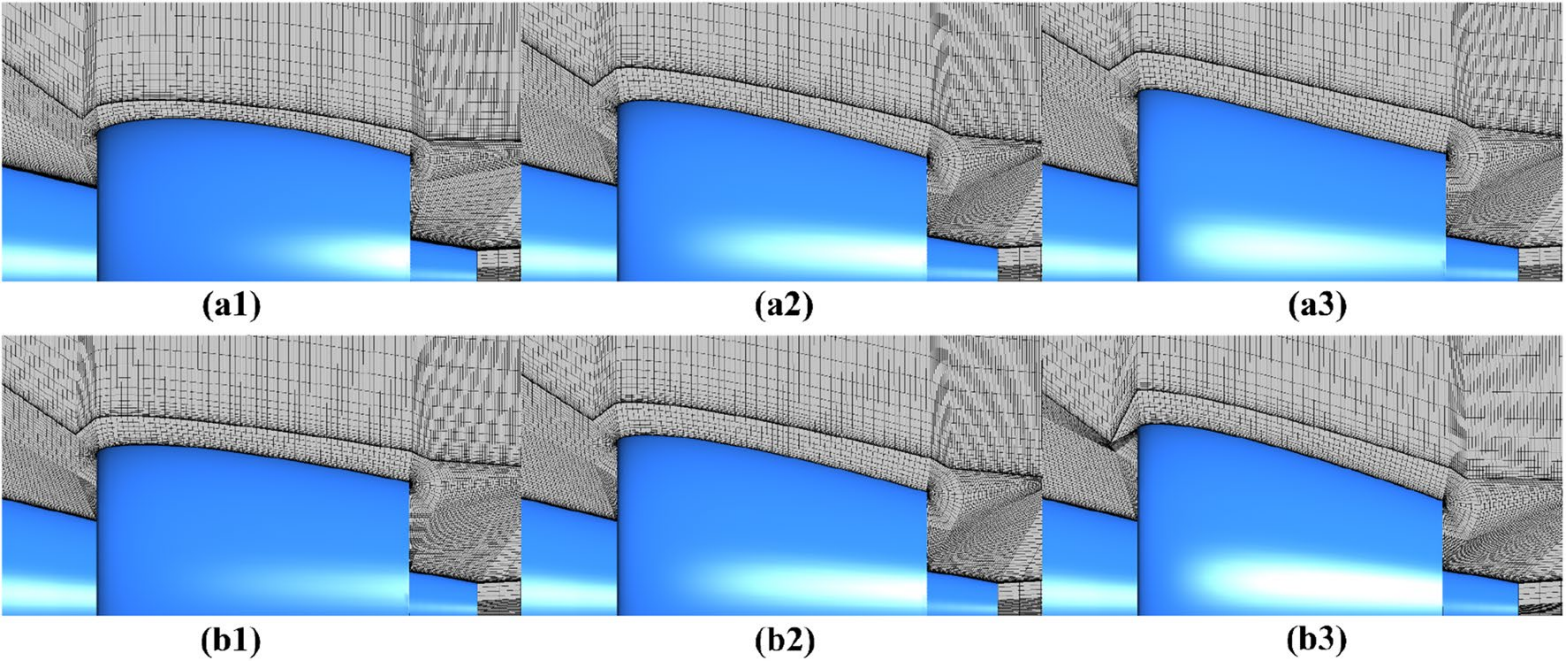
The close-up views of the mesh around the accelerating and decelerating ducts. The mesh for the pump-jets with different cambers (*f* = 0.25*t*, 0, − 0.25*t*) are corresponded to (**a1**–**a3**), and the mesh for the pump-jets with different angles of attack (*α* = − 4°, 0°, 4°) are corresponded to (**b1**–**b3**).

The quality of the computational grid has a direct impact on the accuracy of numerical simulation results and the convergence of the simulation process. While both hexa-structured and hybrid-unstructured grids can yield comparable levels of accuracy during simulation, hexa-structured grids are better suited for detailed flow field simulations. This is due to the compatibility of structured grid with boundary layer calculations and its effectiveness in reducing grid volume^20,21^. For effective mesh generation, partitioning the entire computational domain is advantageous. In line with this, this study employs structured grids generated using ANSYS ICEM CFD for all investigated computational domains. Figure 7 visually displays the structured grids covering the entire computational domain, showcasing the external configuration of the pump-jet propulsion. Additionally, Fig. 7 provides a glimpse of the structured grids within the internal computational domains of the pump-jet, encompassing the pre-stator, rotor, and stator domains. To offer a more detailed view, Fig. 8 presents close-up perspectives of the grid distribution around the ducts of the pump-jets. This includes grid representations for pump-jets featuring various cambers and angles of attack, exemplifying cases such as camber *f* = 0.25t, 0, − 0.25t, and angles of attack *α* = − 4°, 0°, 4°. It is worth noting that the quality of each computational domain grid surpasses 0.42.

To ensure that the prediction accuracy remains unaffected by grid size while also optimizing computational resources and avoiding unnecessary complexity, it becomes imperative to conduct a thorough grid-independence validation of the computational domain. This step involves generating seven different grid schemes, each with varying grid densities, as detailed in Table 2. Subsequently, the primary objective of this comparison is to gauge the influence of varying grid densities on essential parameters like thrust coefficient, torque coefficient, and propulsion efficiency. This assessment is conducted within the context of the overall computational domain under the specific operational condition of *J* = 0.97. Based on the results obtained from these comparisons, a determination can be made regarding the most suitable grid scale that effectively balances precision and computational efficiency.

**Table 2:** Comparison of different quantities of grids for grid independence analysis.

*J*	Number of grids	*KT*	*KQ*	*η*
0.97	3,273,753	0.35914	0.069306	0.803722
	10,814,777	0.369034	0.069638	0.821917
	14,613,462	0.376701	0.069783	0.837259
	15,866,790	0.37259	0.068679	0.841433
	16,605,795	0.374145	0.068729	0.844325
	17,680,826	0.375479	0.068816	0.846268
	19,056,782	0.3755812	0.068852	0.846576

The provided table illustrates a discernible pattern wherein performance parameters initially ascend to a peak with an increase in grid number, subsequently declining before reaching a plateau. Notably, the disparity between propulsion efficiencies diminishes progressively, particularly after the second rise in grid numbers. Therefore, with the objective of attaining heightened accuracy in hydrodynamic performance results and capturing detailed flow field information near the boundaries, all while optimizing computational resources, the case involving 17.68 million grids is deliberately selected for the ensuing numerical investigation within this study.

### Numerical method validation

**Figure 9:**
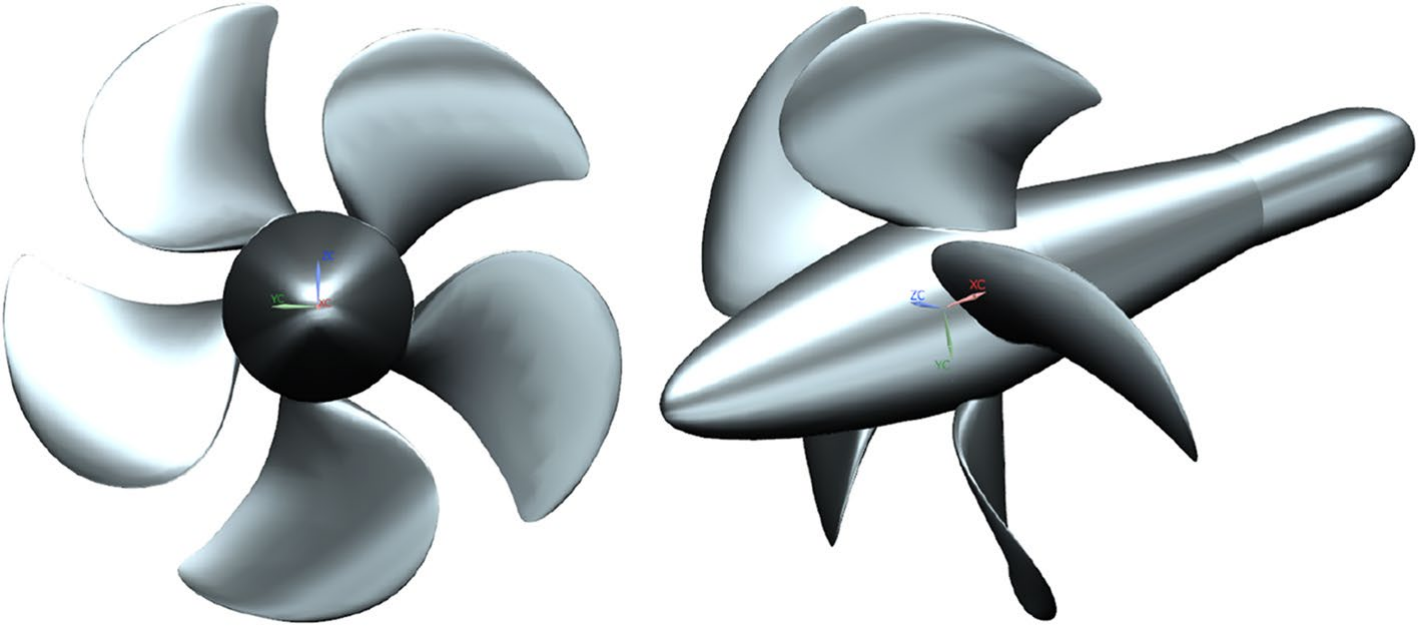
Construction of propeller VP1304.

**Figure 10:**
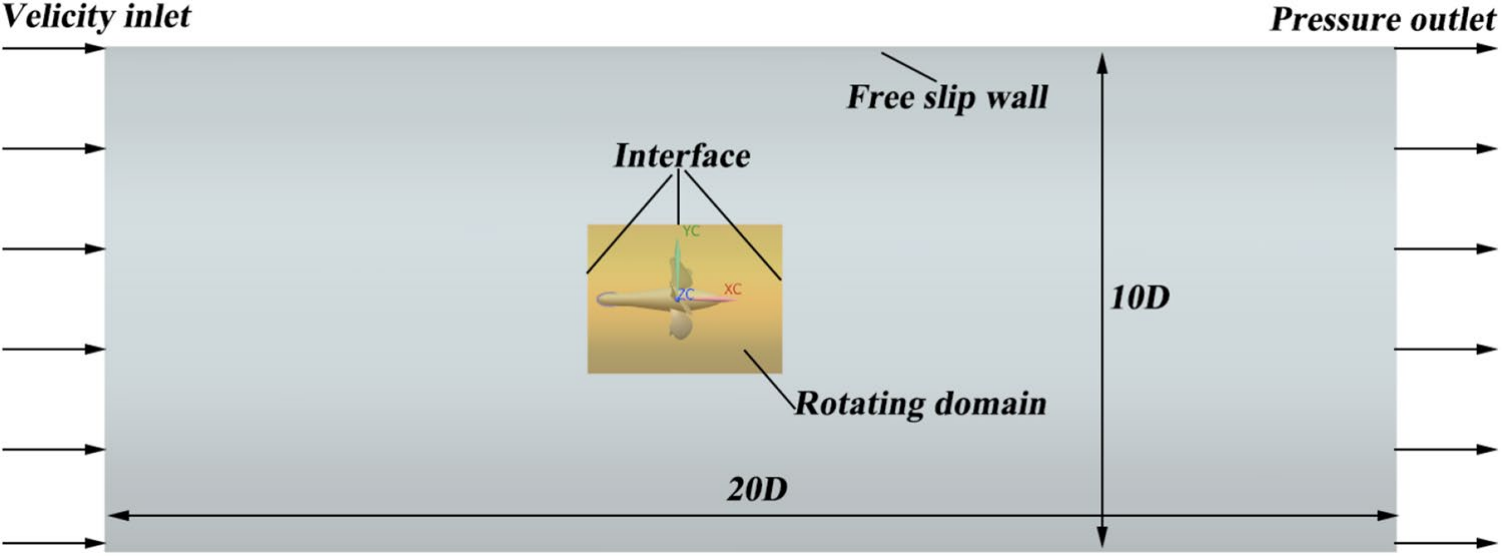
The boundary conditions of VP1304.

**Figure 11:**
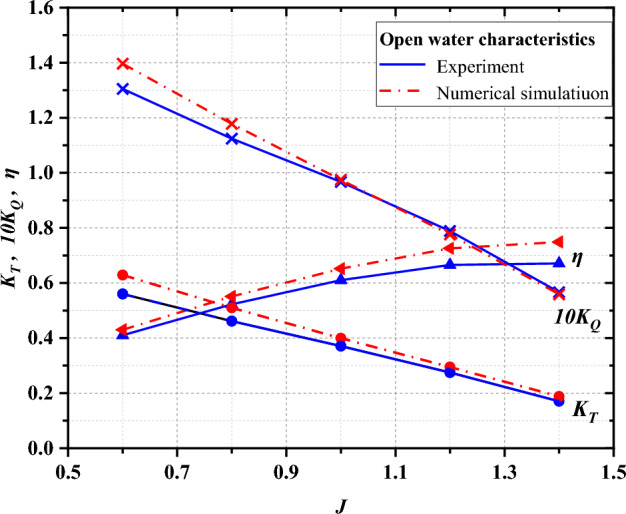
Validation of CFD results with experimental data of SVA.

This study employs the validation of numerical simulation feasibility on propeller VP1304, which features 5 blades and is renowned for being one of the most commonly employed propellers. This selection is attributed to the status of VP1304 as a benchmark test propeller, extensively used for evaluating numerical investigation test cases, as established by SVA. Meanwhile, the pump-jet propeller used in this study is in the same working condition as this propeller, with similar boundary conditions. The significance of propeller is further underscored by its classification as a classic test propeller. The open water experiments were conducted by SVA during the smp’11 workshop, focusing on propeller performance under non-cavitating conditions. Essential geometry and foundational structural parameters are comprehensively outlined in Fig. 9 and Table 3, respectively. Figure 10 provides insight into the boundary conditions of VP1304, revealing the configuration of the computational domain, which encompasses both the external flow field and the rotational domain. Aligning with the present approach of this study, the VP1304 numerical simulation employs the SST *k*–*ω* turbulence model. To ensure robust validation, the hydrodynamic performance coefficients derived from the simulation outcomes are rigorously compared with experimentally measured data from SVA^22^. This comparison, graphically represented in Fig. 11, demonstrates a minor discrepancy of less than 10%, affirming the accuracy of the numerical simulation.

**Table 3:** Main structural parameters of propeller VP1304^22^.

Parameters	Value
Diameter	0.25 m
Hub ratio	0.3
Chord length r/R = 0.7	0.104
Skew	18.837°
Pitch ratio r/R = 0.7	1.64
Area ratio	0.78

**Figure 12:**
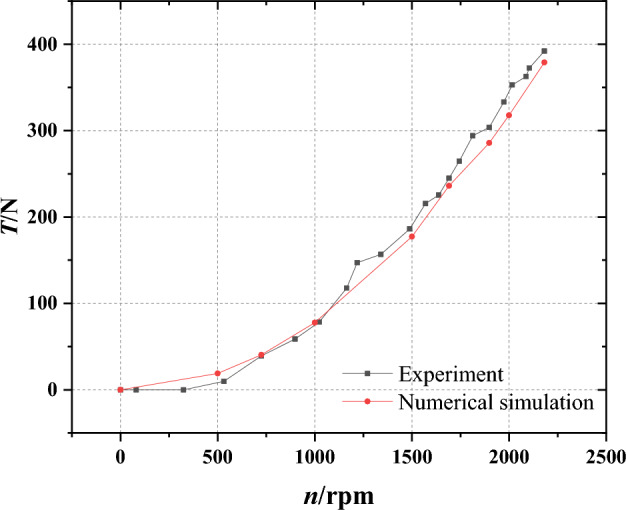
Validation of CFD results with experimental data of pump-jet under condition.

Aiming at further validating the feasibility of numerical investigation, the SST *k*–*ω* turbulence model is also employed to scrutinize the original pump-jet model in this study. Given the constrained nature of the experimental conditions, the pump-jet test is conducted while the system remains moored, which means the experiment is carried out under the condition of no advance rate. In essence, the pump-jet is submerged in a water channel devoid of inflow velocity, with changes solely introduced through adjustments in rotational speed during the experiment. Figure 12 illustrates the comparison between the numerical simulation results and experimental findings, revealing that the maximum error falls within ten percent. This outcome underscores the alignment between the numerical predictions and experimental data, suggesting favorable agreement between the two across the range of pump-jet operation under moored conditions.

The juxtaposition of the aforementioned data sets underscores a key observation: the results derived from the numerical methodology employed in this paper exhibit substantial congruence with the experimental outcomes. This congruence serves as a testament to the stability and reliability of the numerical approach adopted herein, thereby affirming the overall accuracy and validity of this study.

## Results and discussion

This study aims to comprehensively investigate and contrast the impact of accelerating and decelerating ducts on the transient flow behavior of a pump-jet. The analysis entails a combination of steady and unsteady simulations to achieve the flow field characteristics. With the specific research goal in mind, the ensuing sections will delve into the transient outcomes pertaining to varying camber sizes (*f* = 0.5t, 0.25t, 0, − 0.25t, − 0.5t) and different angles of attack (*α* = 4°, 0°, − 4°) of the pump-jet. The quantities of rotor and stator blades are represented by n_r_ and n_s_, respectively. In addition, the total time of the rotational period *T*_n_ is set as 0.025 s, while the corresponding frequency *f*_n_ is set as 40 Hz.

### Open water performance

#### Propulsion performance

**Figure 13:**
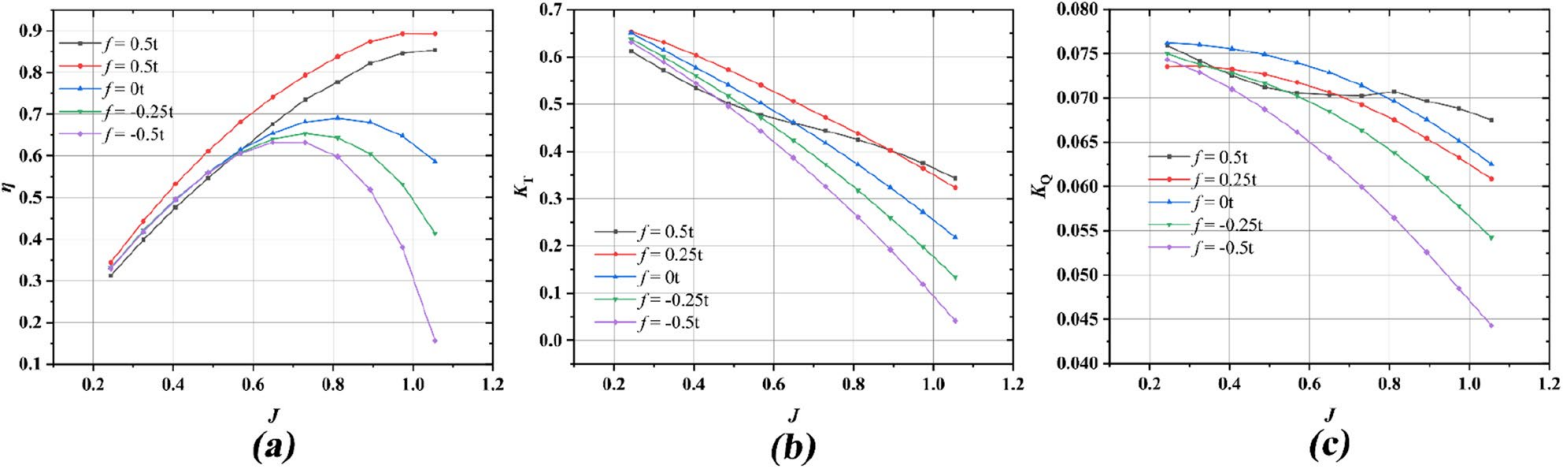
Comparison of propulsion performance for pump-jet with different ducts by changing cambers (*f* = 0.5*t*, 0.25*t*, 0, − 0.25*t*, − 0.5*t*).

**Figure 14:**
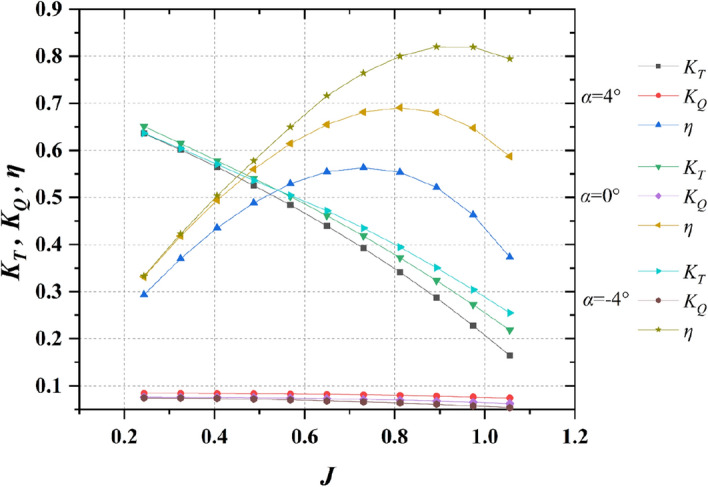
Comparison of propulsion performance for pump-jet with different ducts by changing angles of attack (*α* = − 4°, 0°, 4°).

Figures 13 and 14 present a comprehensive comparison of pump-jet propulsion efficiency, involving ducts featuring varying cambers (0.5t, 0.25t, 0, − 0.25t, − 0.5t) and angles of attack (− 4°, 0°, 4°), across distinct operational conditions ranging from *J* = 0.24 to *J* = 1.06. Meanwhile all the cases maintain a constant rotational speed of *n* = 2400 rpm across all configurations. The curves within Fig. 13 exhibit notable trends. Overall, the peak open water efficiency experiences a modest decline as the camber shifts from positive (*f* = 0.5t) to negative (*f* = − 0.5t). In contrast, the decelerating duct displays sustained high propulsion efficiency across a broader range of advance coefficients, as compared to the accelerating ducts. As the camber increases, the ascent rate of propulsion efficiency becomes more pronounced until it reaches its zenith. This behavior is attributed to the decreasing decline rate of the pump-jet thrust coefficient (*K*_t_) coupled with the relatively minor disparity in torque coefficient (*K*_Q_). Furthermore, the progression towards lower camber values within the duct section causes the peak propulsion efficiency to shift towards lower advance coefficients (*J*). For instance, the location shifts from *η* of 85.40% at *J* = 1.06 (*f* = 0.5t) to *η* of 89.27% at *J* = 0.97 (*f* = 0.25t), and then to *η* of 63.22% at *J* = 0.65 (*f* = − 0.5t). This shift is accompanied by elevated values of *K*_t_, *K*_Q_, and *η*, except for the duct with camber *f* = 0.5t at low advance coefficients. Notably, the duct with camber *f* = 0.5t behaves distinctively from others. It exhibits the lowest thrust and efficiency values at small advance coefficients, yet surpasses all accelerating ducts at higher advance coefficients due to its remarkable efficiency ascent rate and slower thrust coefficient descent rate. Comparatively, the torque coefficient curves exhibit marginal variations across the advance coefficient spectrum, whereas the thrust coefficient curves demonstrate higher sensitivity, factoring in the temporal dimension.

Figure 14 provides a visualization of the performance trends exhibited by pump-jet propulsion with varying angles of attack for the adopted ducts, bearing a resemblance to the patterns seen in Fig. 13. This similarity is especially pronounced given that changes in angles of attack do not significantly alter the shape of the duct profile. The simplification of working conditions facilitates a more direct and evident trend for the impact of angles of attack on decelerating and accelerating ducts. Notably, pump-jets featuring ducts with *α* = − 4° and *α* = 0° attain their highest efficiencies of 81.98% at *J* = 0.89 and 69.07% at *J* = 0.81, respectively. In contrast, the pump-jet equipped with a duct at *α* = 4° achieves its maximum efficiency of 56.34% at *J* = 0.63. The consistency in results between the curves in Figs. 13 and 14 highlights the prevailing influence of advance coefficient *J*. This suggests that for decelerating ducts, higher values of *J* yield greater thrust coefficient *K*_t_, thereby magnifying the overall performance of pump-jets equipped with decelerating ducts. The trend is further accentuated by the capability of decelerating ducts to sustain high propulsion efficiency across an extended range of advance coefficients. Furthermore, owing to the comparatively gradual decline in *K*_t_ for pump-jets with decelerating ducts, the rate of increase in *η* becomes more pronounced as the efficiency approaches its peak. This observation underscores the intricate relationship between thrust coefficient behavior and the rising efficiency profile.

#### Unsteady thrust of rotor and stator

**Figure 15:**
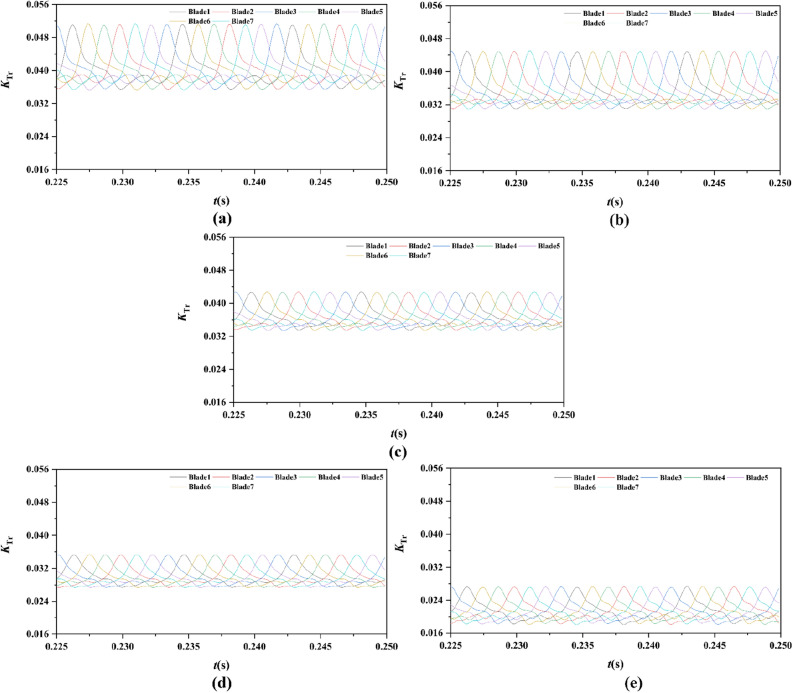
The time domain curves of thrust per rotor blade at *J* = 0.97 under different duct profile cambers, (**a**) *f* = 0.5t, (**b**) *f* = 0.25t, (**c**) *f* = 0, (**d**) *f* = − 0.25t, (**e**) *f* = − 0.5t.

**Figure 16:**
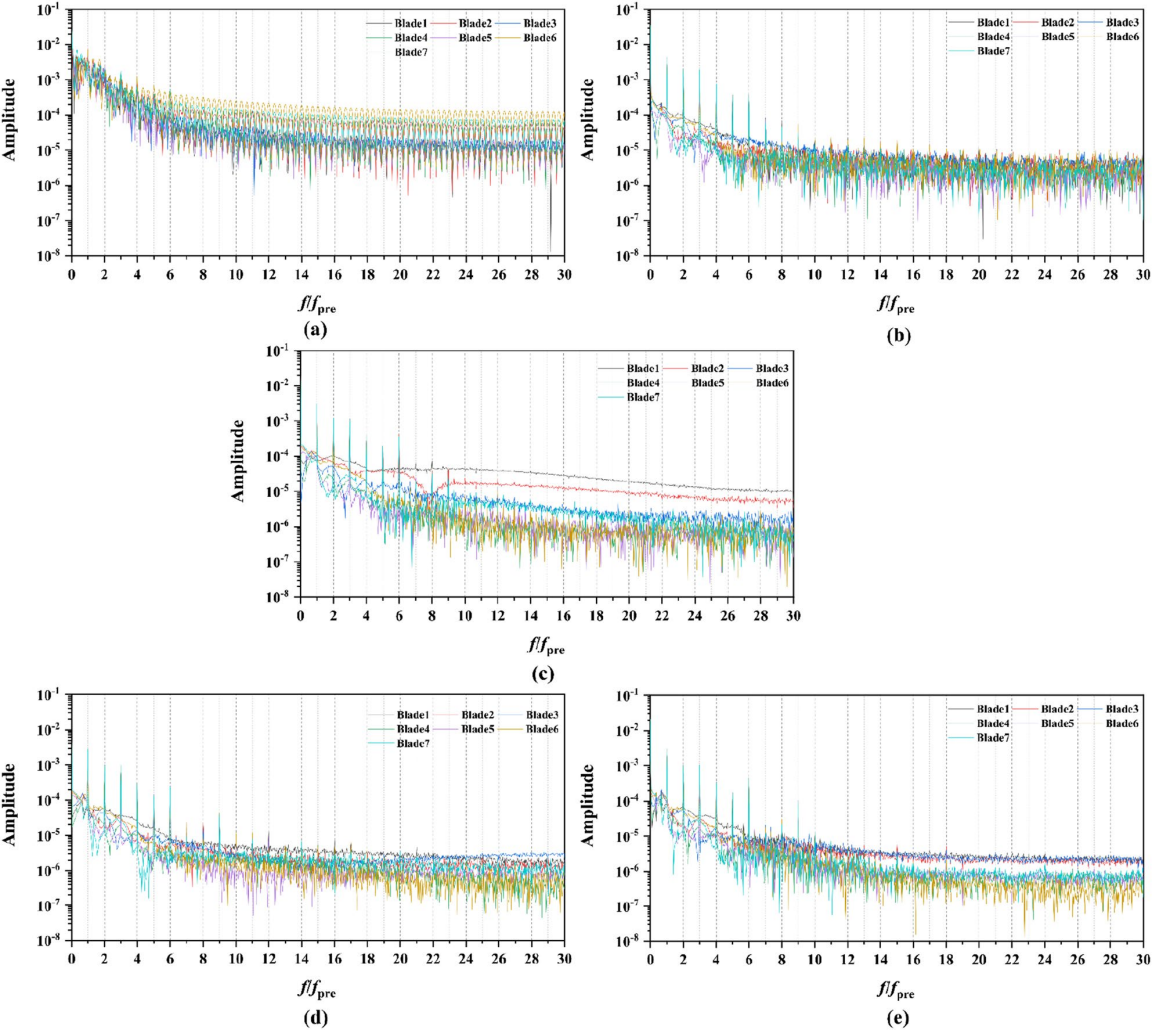
The frequency domain curves of per rotor blade thrust at *J* = 0.97 under different duct profile cambers, (**a**) *f* = 0.5t, (**b**) *f* = 0.25t, (**c**) *f* = 0, (**d**) *f* = − 0.25t, (**e**) *f* = − 0.5t.

**Figure 17:**
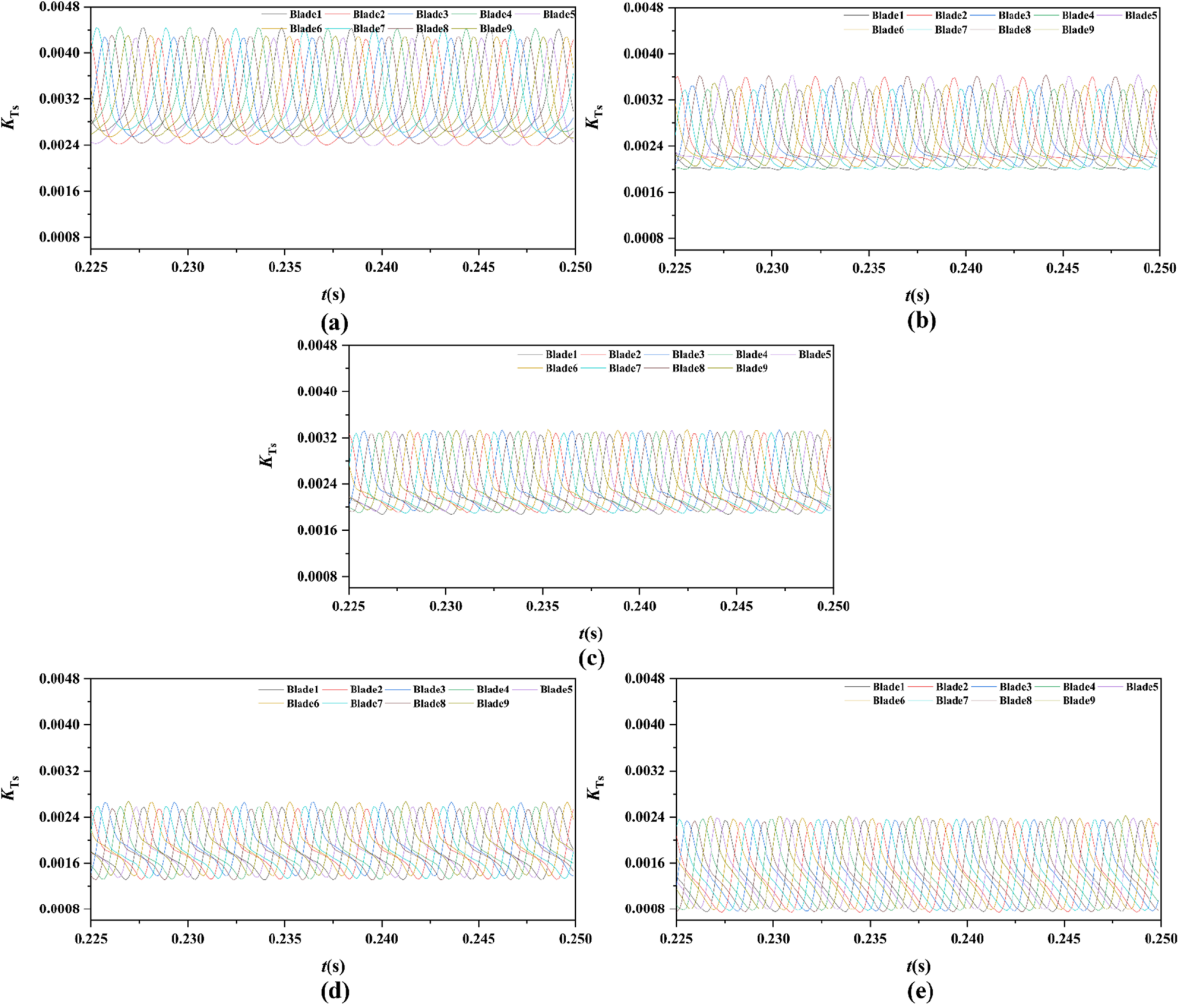
The time domain curves of thrust per stator blade at *J* = 0.97 under different duct profile cambers, (**a**) *f* = 0.5t, (**b**) *f* = 0.25t, (**c**) *f* = 0, (**d**) *f* = − 0.25t, (**e**) *f* = − 0.5t.

**Figure 18:**
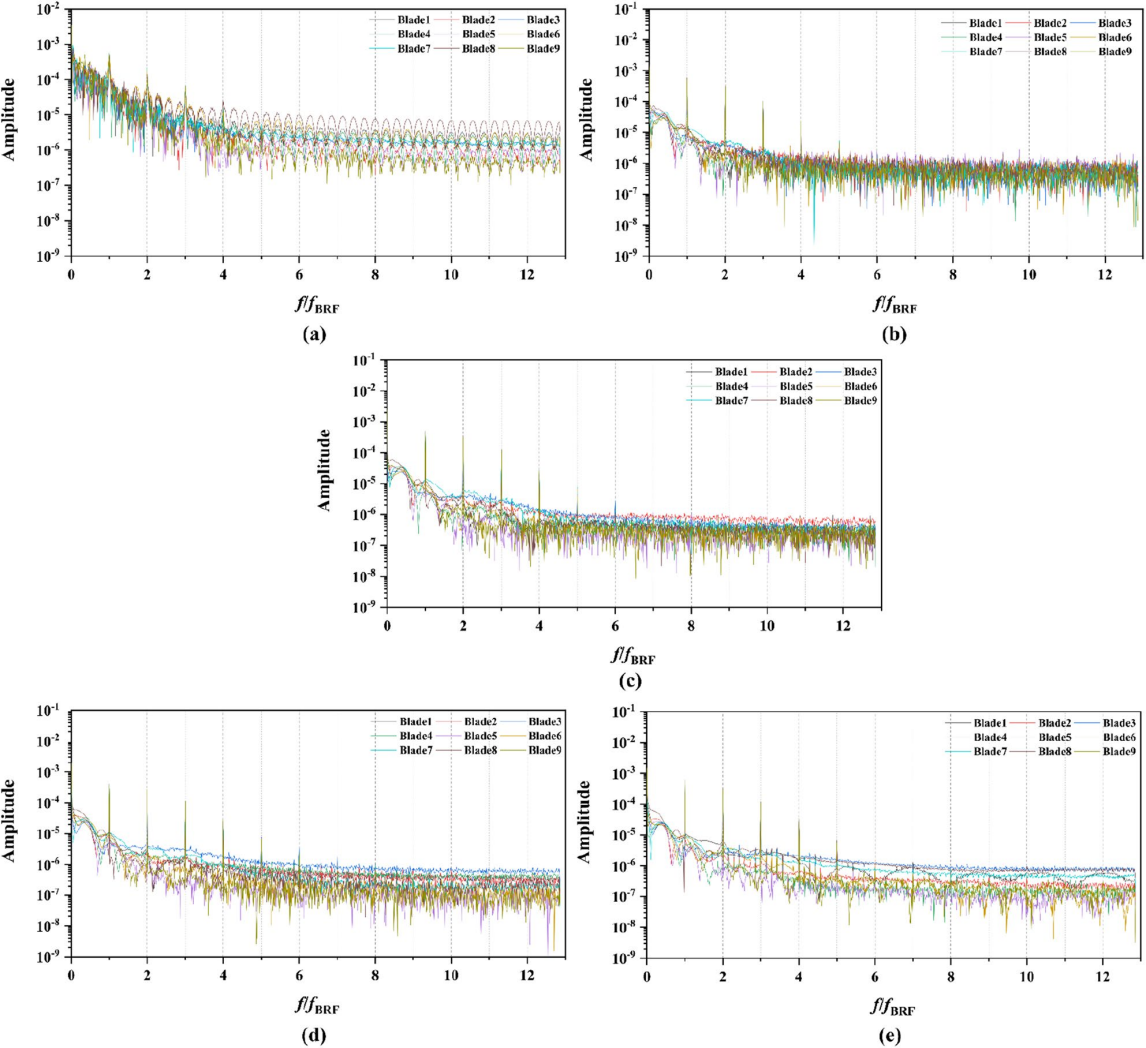
The frequency domain curves of thrust per stator blade at *J* = 0.97 under different duct profile cambers, (**a**) *f* = 0.5t, (**b**) *f* = 0.25t, (**c**) *f* = 0, (**d**) *f* = − 0.25t, (**e**) *f* = − 0.5t.

**Figure 19:**
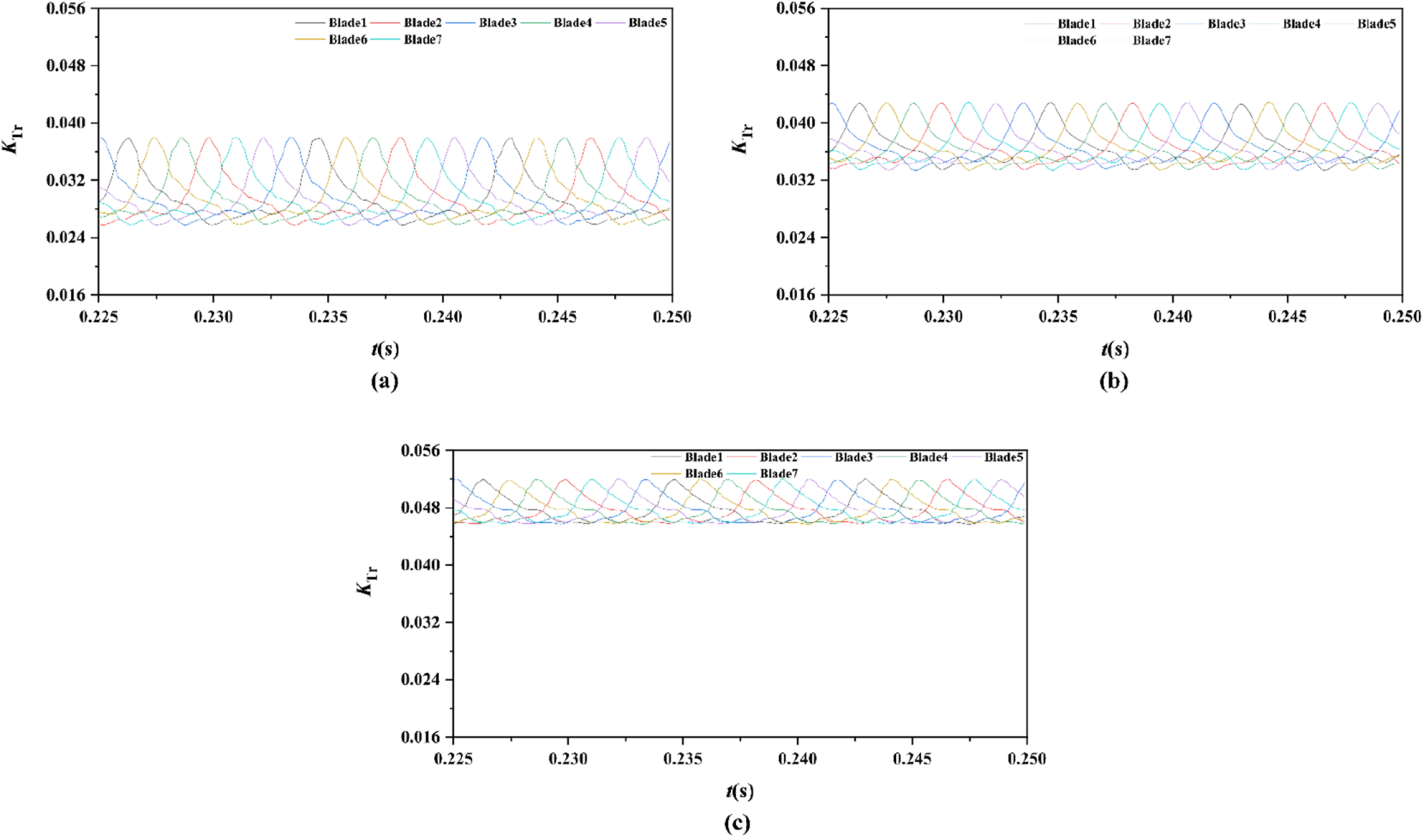
The time domain curves of thrust per rotor blade at *J* = 0.97 under different duct profile angles of attack, (**a**) *α* = − 4°, (**b**) *α* = 0°, (**c**) *α* = 4°

**Figure 20:**
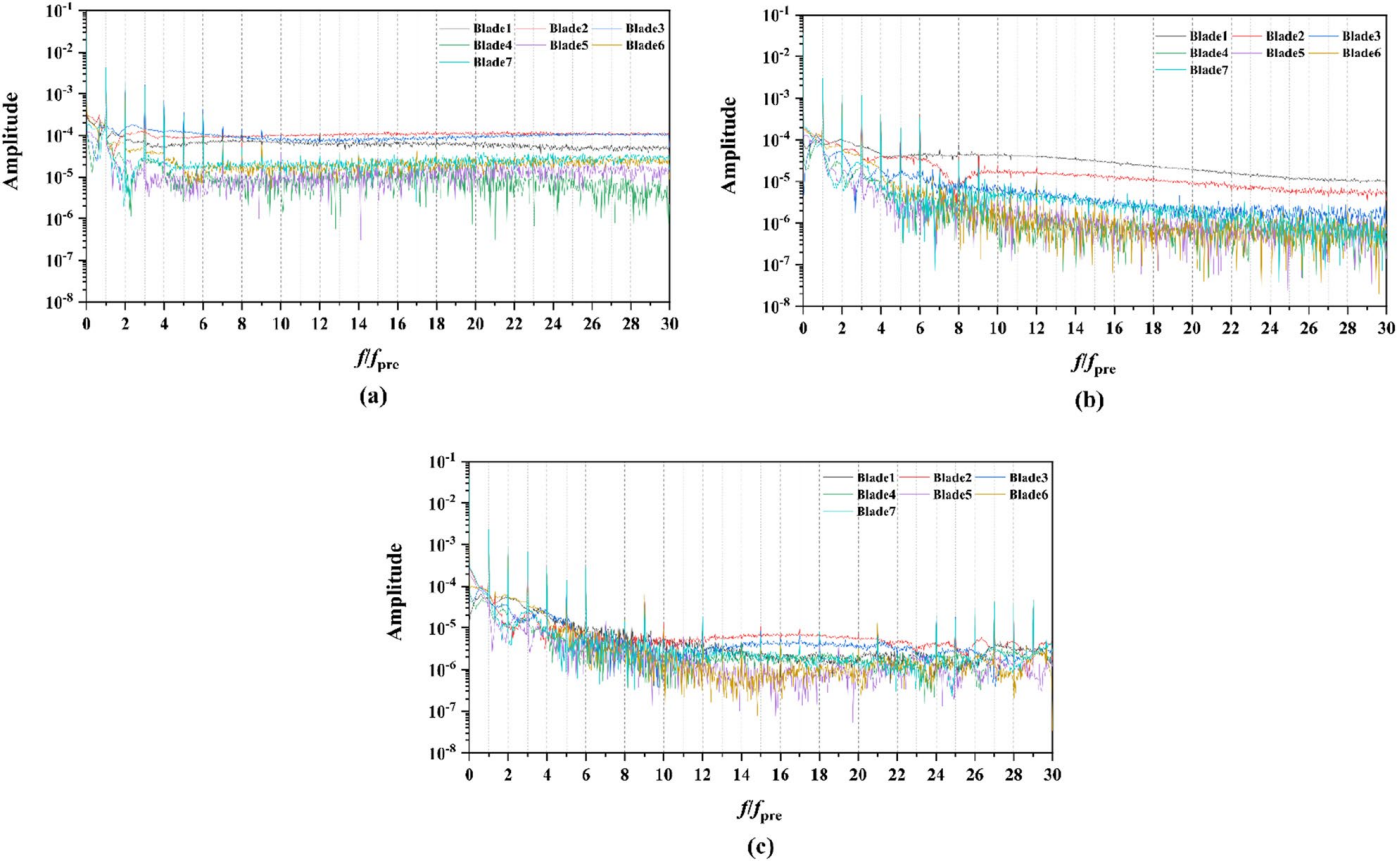
The frequency domain curves of thrust per rotor blade at *J* = 0.97 under different duct profile angles of attack, (**a**) *α* = − 4°, (**b**) *α* = 0°, (**c**) *α* = 4°

**Figure 21:**
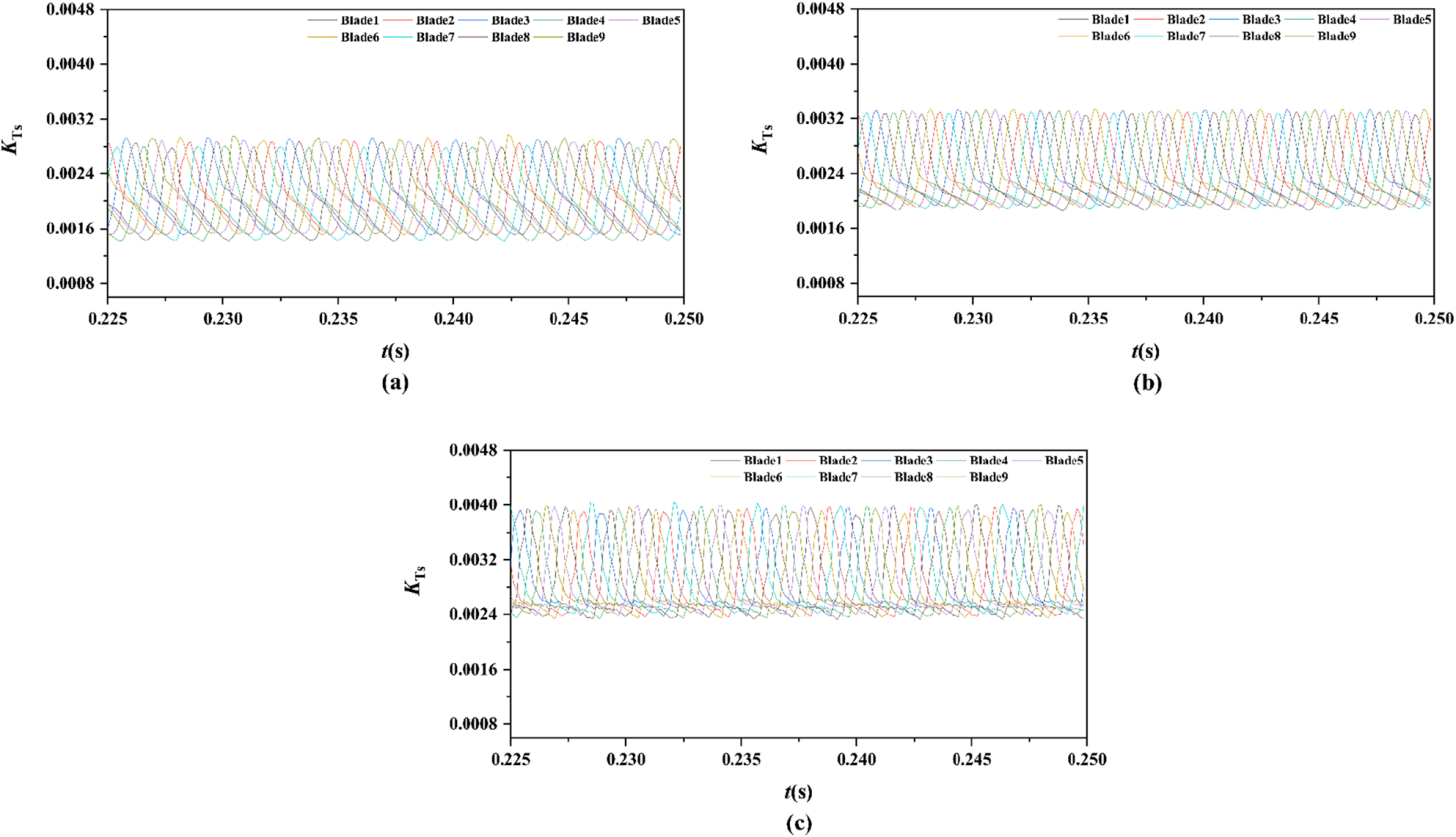
The time domain curves of thrust per stator blade at *J* = 0.97 under different duct profile angles of attack, (**a**) *α* = − 4°, (**b**) *α* = 0°, (**c**) *α* = 4°

**Figure 22:**
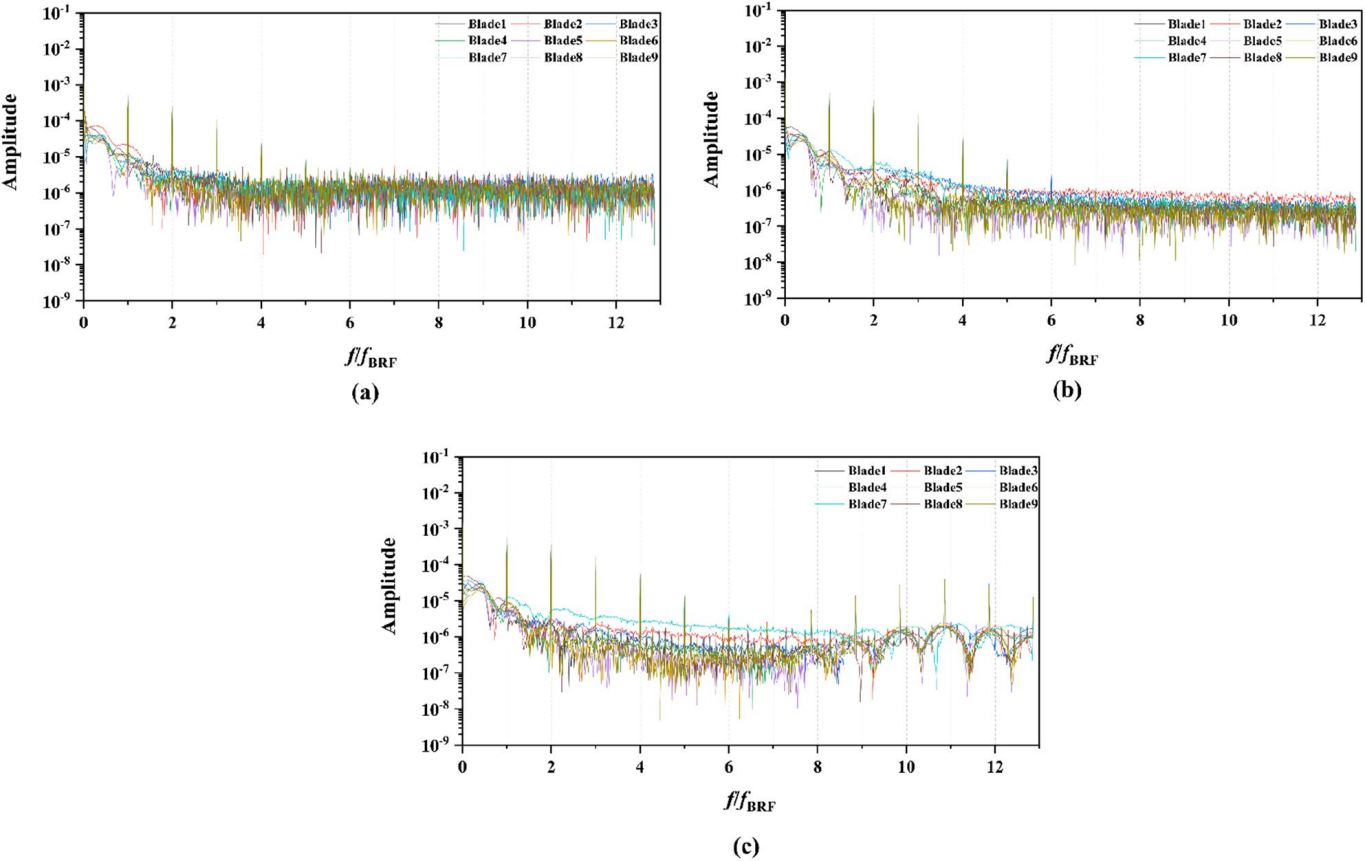
The frequency domain curves of thrust per stator blade at *J* = 0.97 under different duct profile angles of attack, (**a**) *α* = − 4°, (**b**) *α* = 0°, (**c**) *α* = 4°

From Figs. 15, 16, 17, 18, 19, 20, 21 and 22, the time domain and frequency domain curves of each blade transient thrust coefficient of pump-jet with accelerating and decelerating ducts at the design working point *J* = 0.97 are indicated. The thrust coefficient of the pump-jet rotor and stator is made up of each blade thrust of the rotor and stator, respectively. The *K*_T_ curves composed of *K*_Tr_ and *K*_Ts_ are periodic fluctuant, which means that per blade works in a periodic flow field. Figures 15 and 17 show the general tendency of rotor and stator transient force per blade in the time domain on the pump-jets with different duct cambers and angles of attack in one period. Similarly, the time domain curves of the pump-jets with different attack angles are exhibited in Figs. 19 and 21. The variations of *J* = 0.97 for rotor blade curves has a period of 1/3 T (where T is the rotation period of the hub: 1/40 s = 0.025 s), whereas for stator blade curves, a different period of 1/7 T is observed. It is found that the time domain curves of per rotor blade transient thrust coefficient own three peaks and three troughs relative to the quantity of pre-stator blade, caused by the pre-swirl influence of the pre-stator on the inflow. Meanwhile, the periodic influence, seven peaks and seven troughs appeared on the transient thrust coefficient curves per stator blade which is equal to the rotor blade number. Additionally, the peak areas per rotor blade are broader than that per stator blade, and areas of gentle descent after the peak of each curve, induced by the tip clearance flow and the distortion. The range of fluctuation on the rotor blade is more severe than that on the stator blade.

In Fig. 15, the comparative results are discussed by the subtitles (a)–(e), corresponding to the transient force per rotor blade of pump-jet with duct *f* = 0.5t, 0.25t, 0, − 0.25t, − 0.5t, respectively. The results indicate that lower *f* leads to the decrease of values of the thrust peak and trough. Meanwhile, the decline of *f* not only reduces the average value of unsteady force, but also lowers the differences in thrust coefficient values between the peak and trough of curves. On the other hand, Fig. 17 shows the comparative time domain curves of unsteady thrust per stator blade of pump-jet with duct cambers. The time-domain curves demonstrate the similar tendency as described in the transient force per rotor blade. Additionally, the magnitude of the fluctuations shows an upward trend along with the increment of *f*, except for the situation of pump-jet with *f* = 0.5t and − 0.5t.

For the time domain curves of pump-jets with different angles of attack, the curve patterns are shown in Fig. 19. The comparative results indicate that the increase *α* improves the average value of unsteady rotor force, whereas decays the differences in rotor thrust coefficient values between the peak and trough of curves. As shown in Fig. 21, the average value of unsteady stator force is also increased, but the minor increment appears in the magnitude of the fluctuations of stator blades.

From the concern of Figs. 16, 18, 20 and 22, the frequency domain curves of thrust coefficients, which are adopted to study the frequency characteristics of the unsteady force, are transformed from the time domain curves through the method of Fast Fourier transform (FFT) at all the advance coefficients. The frequency domain spectrum of per rotor blade thrust coefficient has the same peaks in *nf*_pre_ (*n* = 1, 2, 3,…, and *f*_pre_ = *n*_pre_*f*_n_ is the pre-stator blades passing frequency), while for per stator blade thrust coefficient, the frequency domain amplitude exhibits the same peaks in *nf*_BRF_ (*n* = 1, 2, 3,…, and *f*_BRF_ = *n*_rotor_*f*_n_ is the pre-stator blades passing frequency). It is found that per rotor and stator blade amplitude of direct-current (DC) which is at the zero frequency is equal to the periodic averaged values of per rotor and stator blade, respectively. In terms of the loading of rotor blade, the amplitude at the blade passing frequency is about 8.5% of the direct-current component, and about 5.1% at double times *f*_pre_. Moreover, the amplitude becomes lower at the high order of the harmonic. Compared to the frequency domain curves of rotor blade, the peak value of the stator blade tends to decay more slowly, that is 16.4% of the direct-current component at the blade passing frequency and 6% at the double times of the blade passing frequency. Except for the direct-current components, the main parts of the rotor and stator blade thrust are made up of the amplitudes at peaks, which are prone to the vibration and fluctuation.

In Fig. 16, the frequency domain curves are compared by the subtitles (a)–(e), corresponding to the pump-jet with different duct cambers. It is demonstrated that increasing *f* can achieve the improvement of direct-current component value and the averaged amplitude value at overall harmonic. The amplitudes at peaks contribute the major components of thrust coefficients of the blade, while the increment of curve amplitude stands for the enhancement of thrust and torque as the fluctuation and time-averaged values are improved, simultaneously. Therefore, the value of pump-jet *K*_t_ becomes higher with the increment of *f*, which is consistent with the regularity shown in Fig. 13. Except for the camber *f* = 0.5t and − 0.5t, the values at curves peaks are decayed more rapid as the camber becomes smaller. Additionally, the minimum difference between different rotor blades is occurred in pump-jet with *f* = 0.25t, which indicates that the internal flow is the most stable in this situation. Figure 18 shows the comparative frequency domain curves of per stator blade of pump-jets with different duct cambers. With the reduction of *f*, the values of peaks are decayed more slowly. Meanwhile, the direct-current component value presents a distinct tendency of descent as the camber decreases, as well as the averaged amplitude value at overall harmonic.

For the frequency domain curves of pump-jets with different angles of attack, Fig. 20 and 22 is consistent with the rule of accelerating and decelerating ducts in Fig. 16 and 18. It is indicated from Fig. 20 that the reduction of amplitude at peaks and the averaged value is obtained by the increasing *α*, which means that the increasing *α* leads to the recline of rotor thrust coefficients. In addition, the difference between different rotor blades occurred less obviously, while the more obvious peaks appear at the high order of the harmonic. In Fig. 22, the curves pattern shows the similar tendency with that in frequency domain curves of rotor blades. Furthermore, the difference between different stator blades and the fluctuation becomes intenser as the attack angle increase, implied that the internal flow field between rotor and stator performs the enhanced instability with the increment of *α*.

#### The pulsating pressure

**Figure 23:**
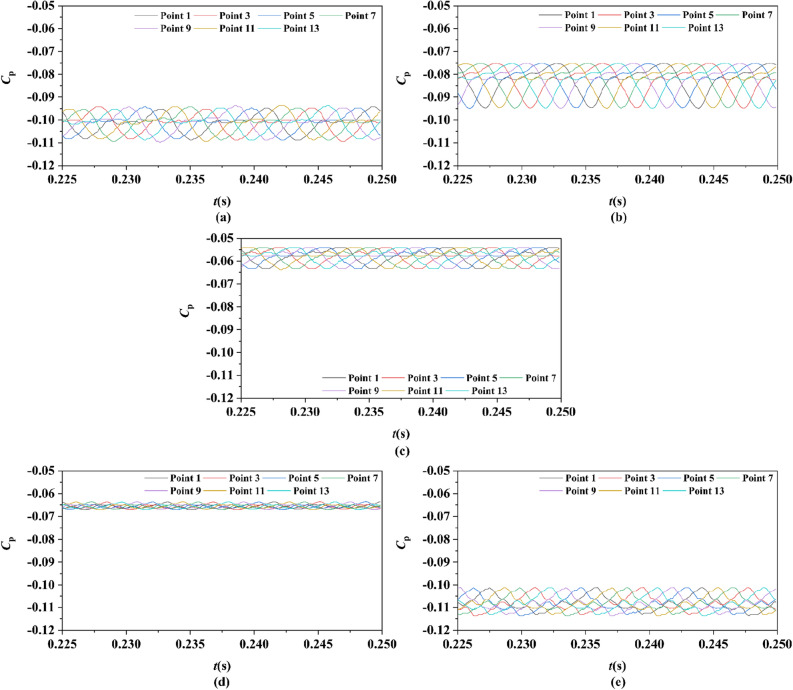
Time history characteristics of Group 1 monitoring points in one period at the tip-clearance region of pump-jets with different *f* (from (**a**) to (**e**): *f* = 0.5t, *f* = 0.25t, *f* = 0, *f* = − 0.25t and *f* = − 0.5t).

**Figure 24:**
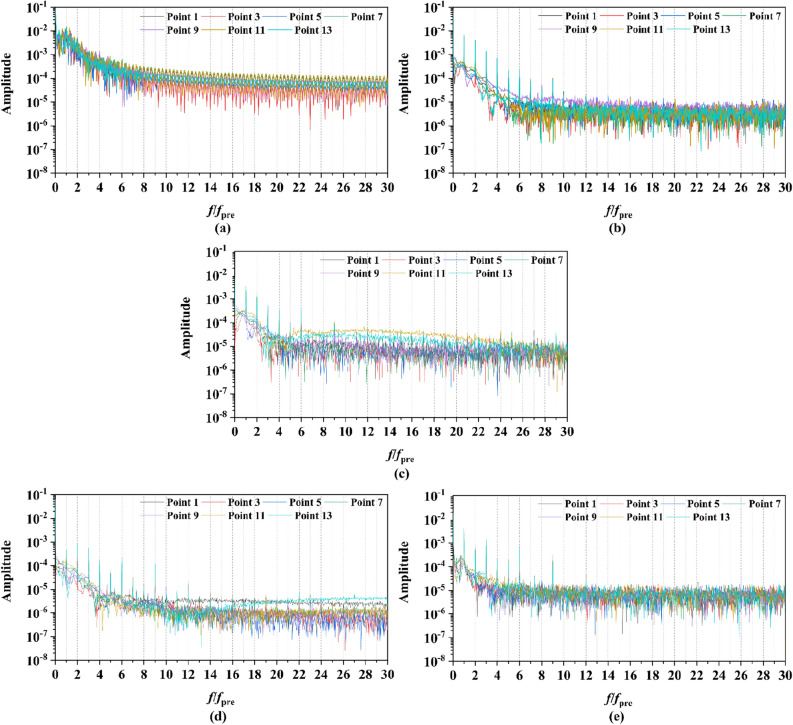
The frequency spectrum characteristics of Group 1 monitoring points in one period at the tip-clearance region of pump-jets with different *f* (from (**a**) to (**e**): *f* = 0.5t, *f* = 0.25t, *f* = 0, *f* = − 0.25t and *f* = − 0.5t).

**Figure 25:**
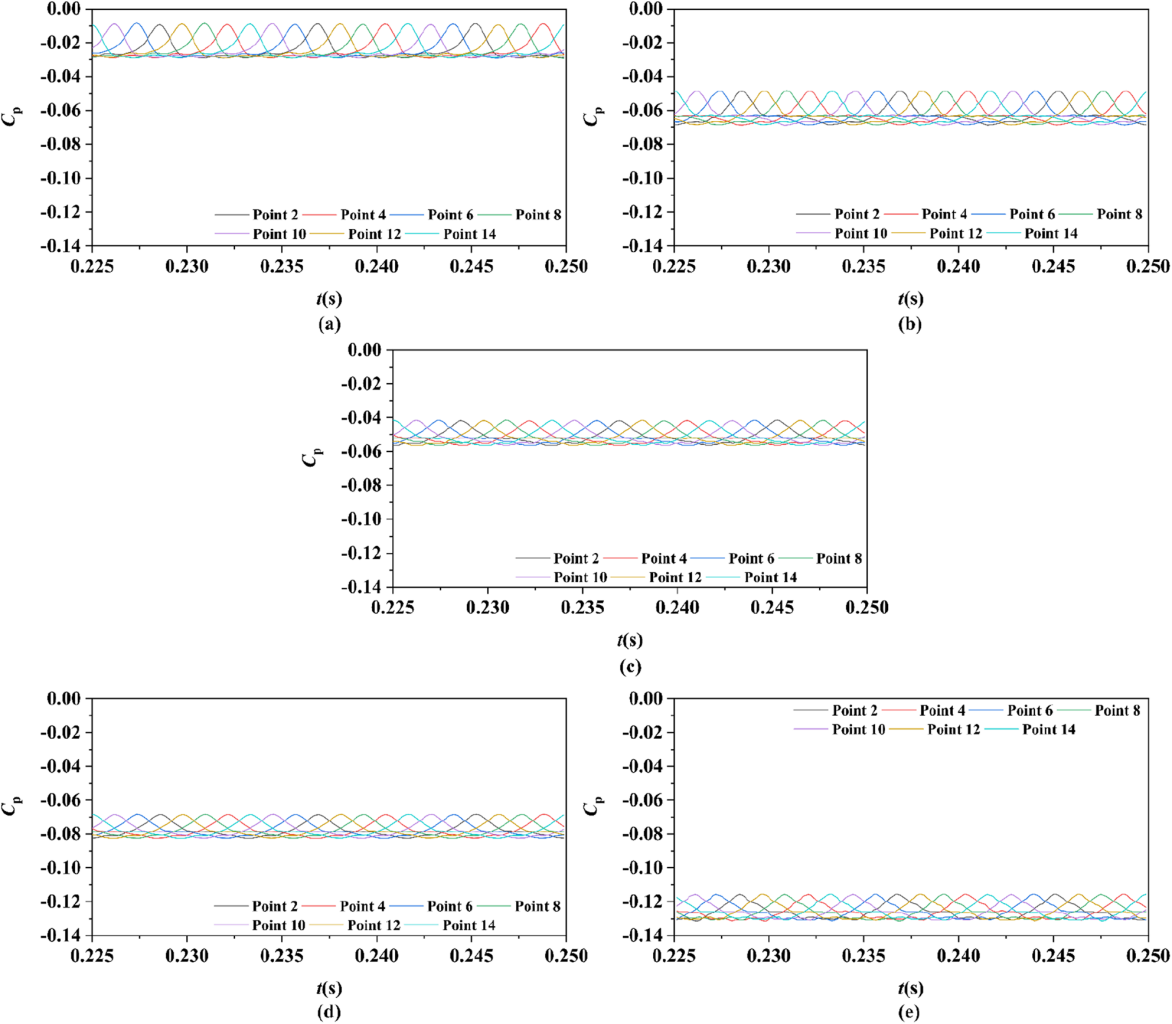
Time history characteristics of Group 2 monitoring points in one period at the tip-clearance region of pump-jets with different f (from (**a**) to (**e**): *f* = 0.5t, *f* = 0.25t, *f* = 0, *f* = − 0.25t and *f* = − 0.5t).

**Figure 26:**
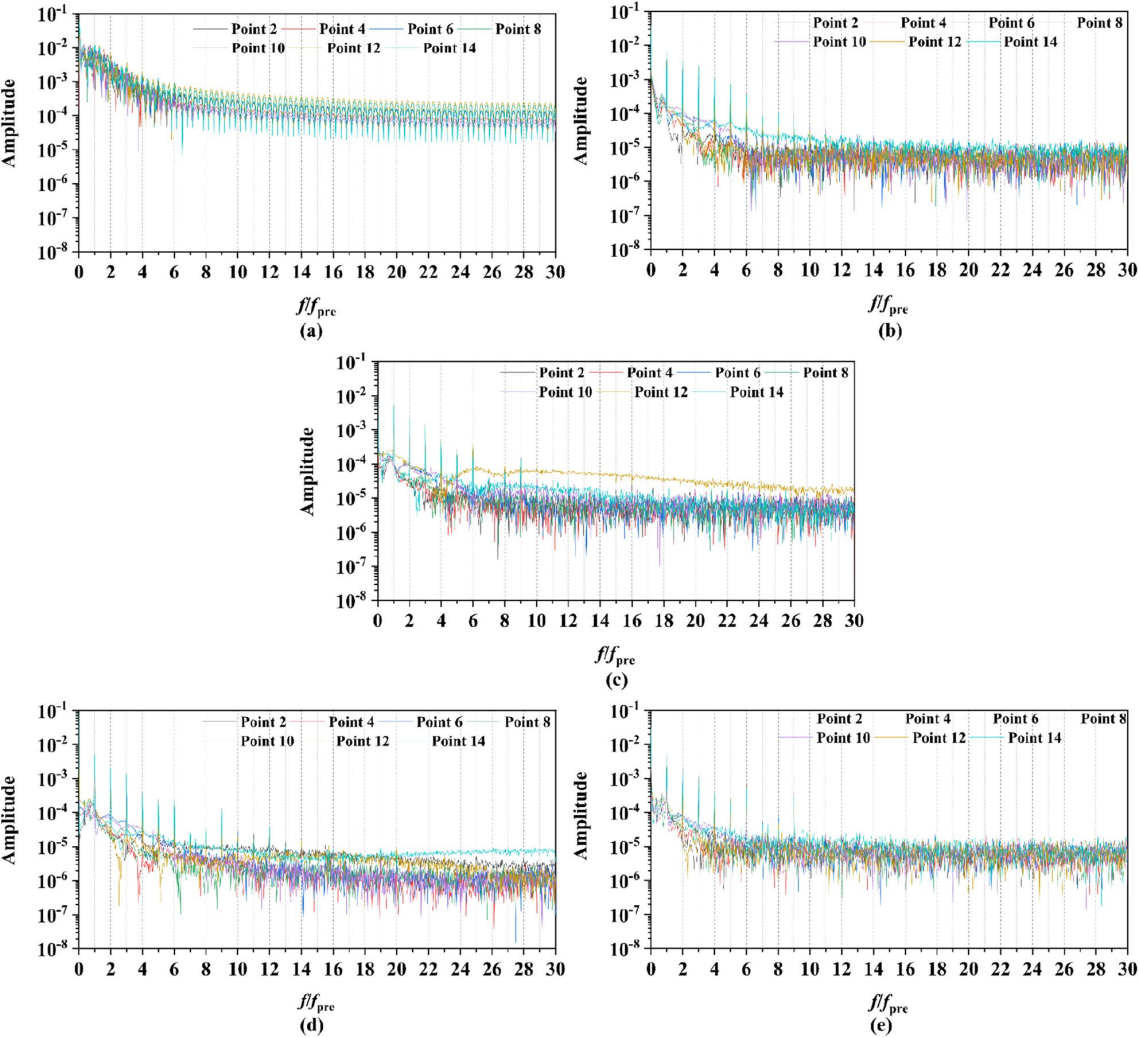
The frequency spectrum characteristics of Group 2 monitoring points in one period at the tip-clearance region of pump-jets with different f (from (**a**) to (**e**): *f* = 0.5t, *f* = 0.25t, *f* = 0, *f* = − 0.25t and *f* = − 0.5t).

**Figure 27:**
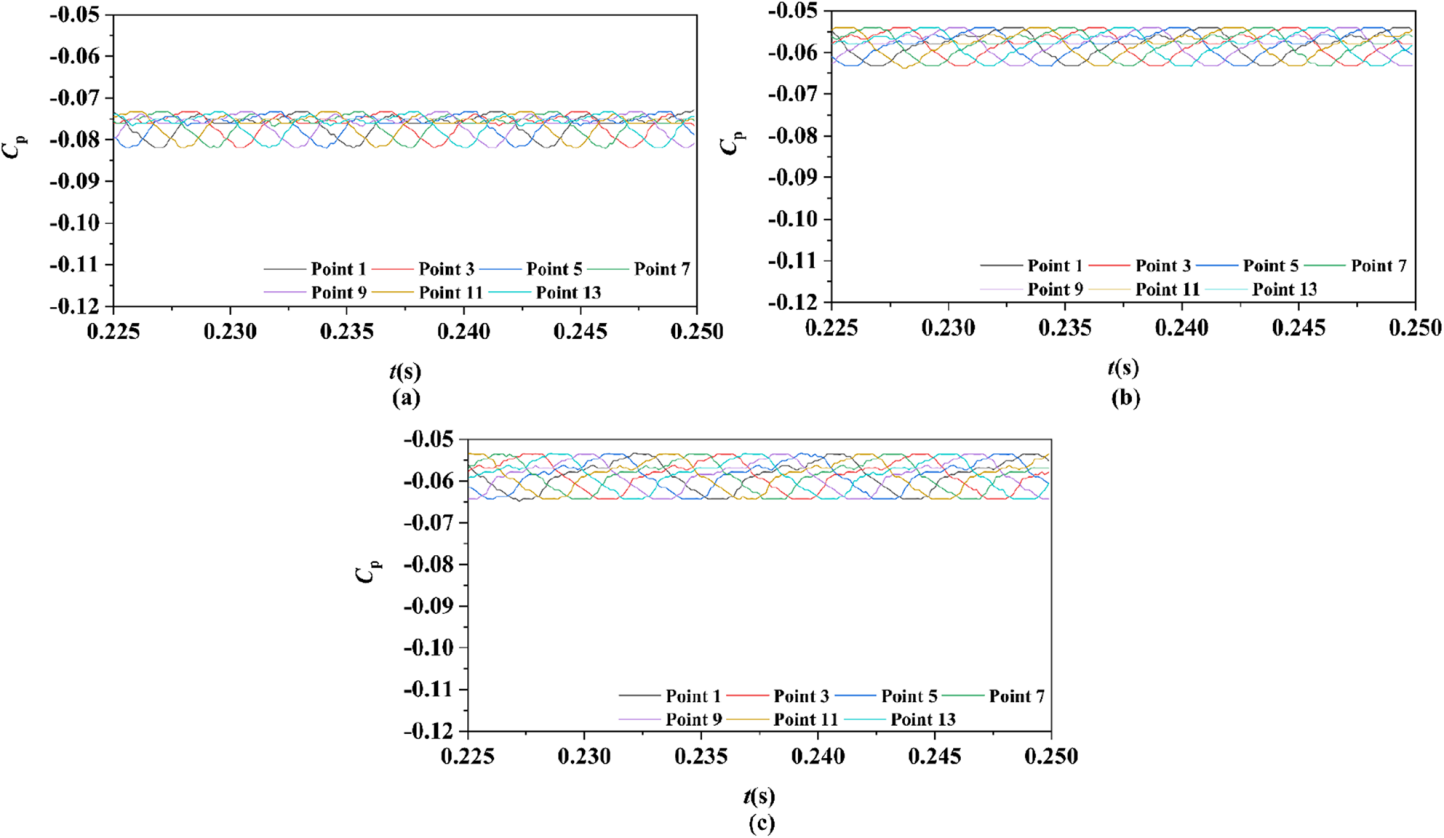
Time history characteristics of Group 1 monitoring points in one period at the tip-clearance region of pump-jets with different f (from (**a**) to (**e**): *α* = − 4°, *α* = 0° and *α* = 4°).

**Figure 28:**
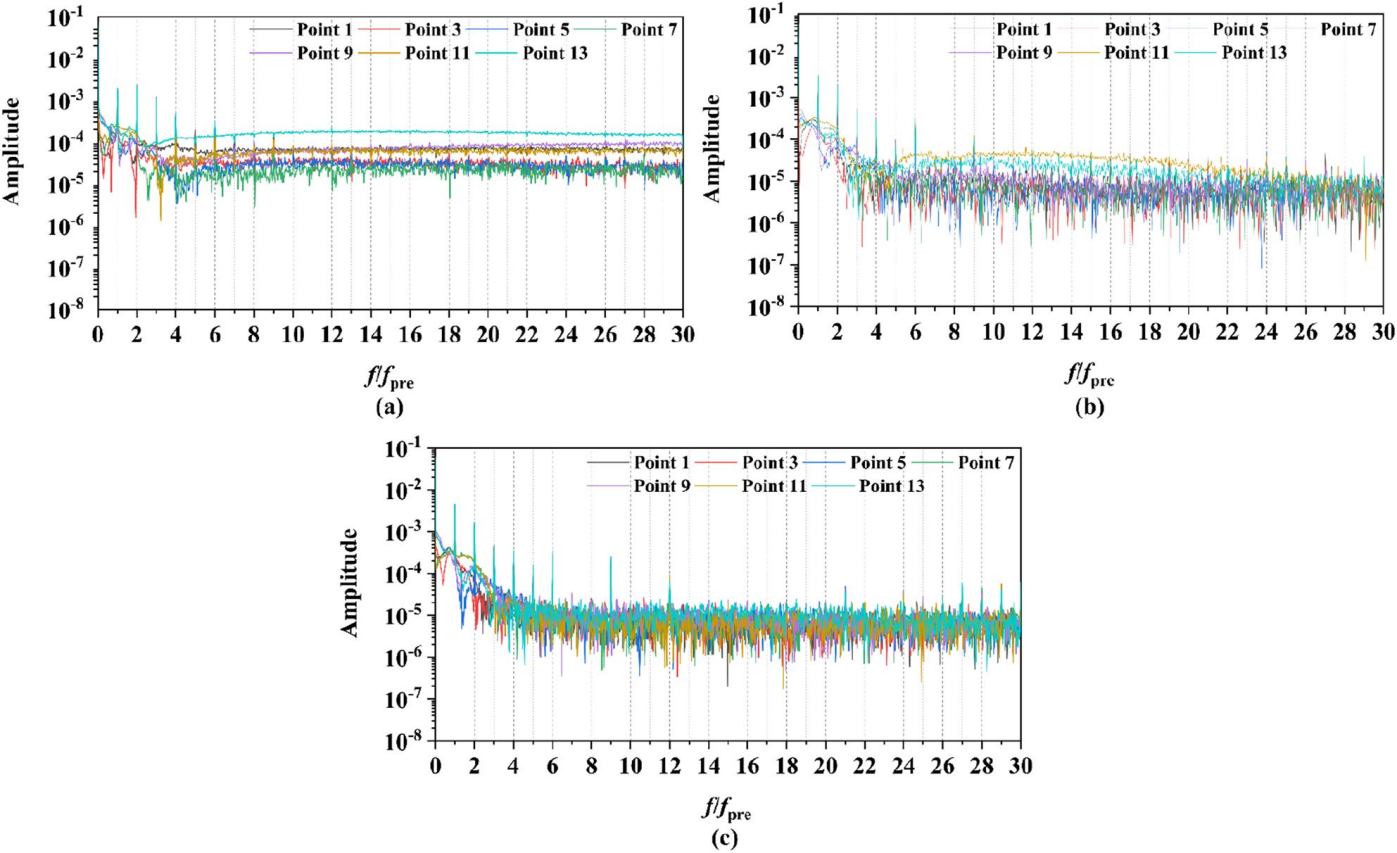
The frequency spectrum characteristics of Group 1 monitoring points in one period at the tip-clearance region of pump-jets with different f (from (**a**) to (**e**): *α* = − 4°, *α* = 0° and *α* = 4°).

**Figure 29:**
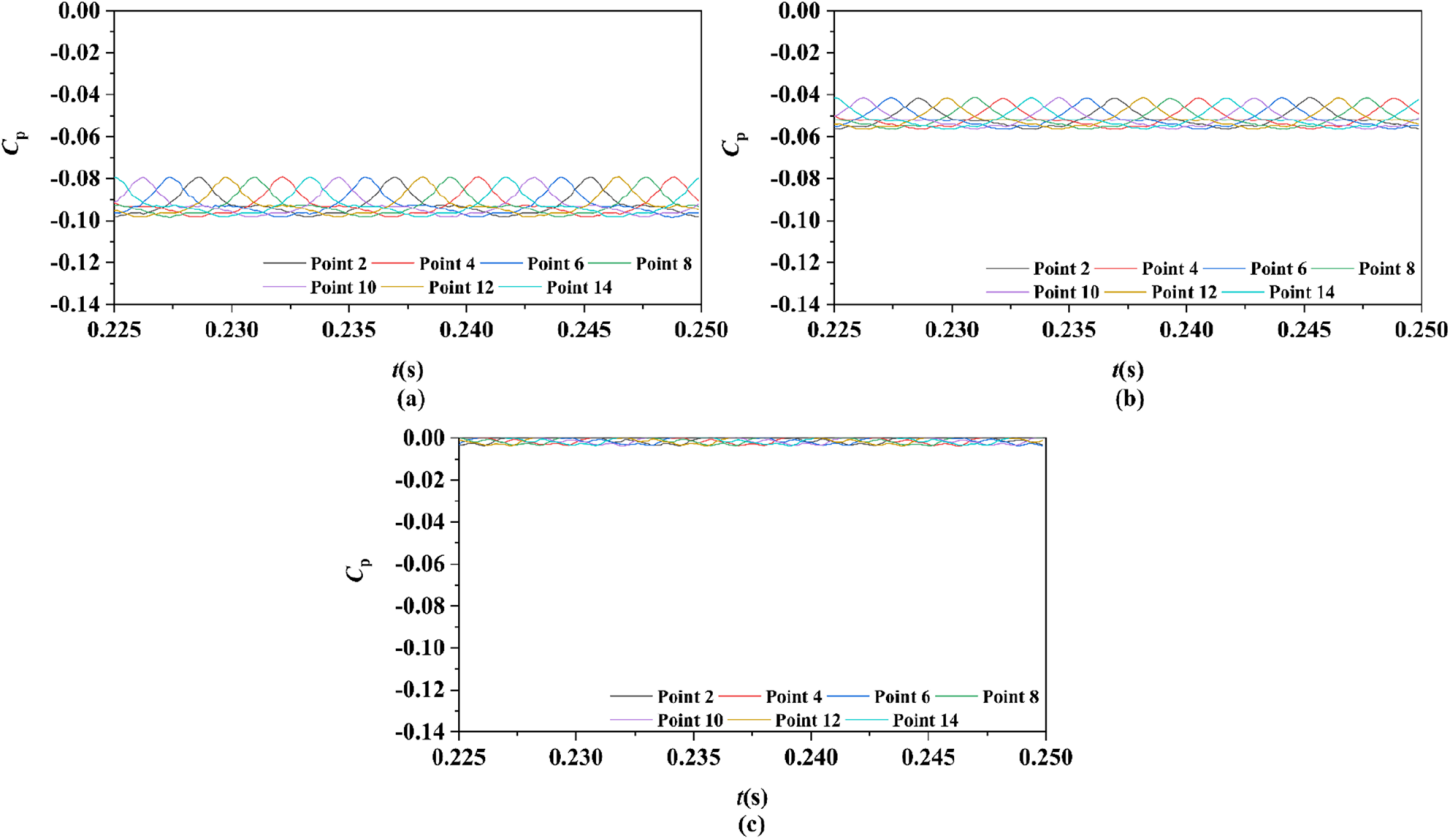
Time history characteristics of Group 2 monitoring points in one period at the tip-clearance region of pump-jets with different f (from (**a**) to (**e**): *α* = − 4°, *α* = 0° and *α* = 4°).

**Figure 30:**
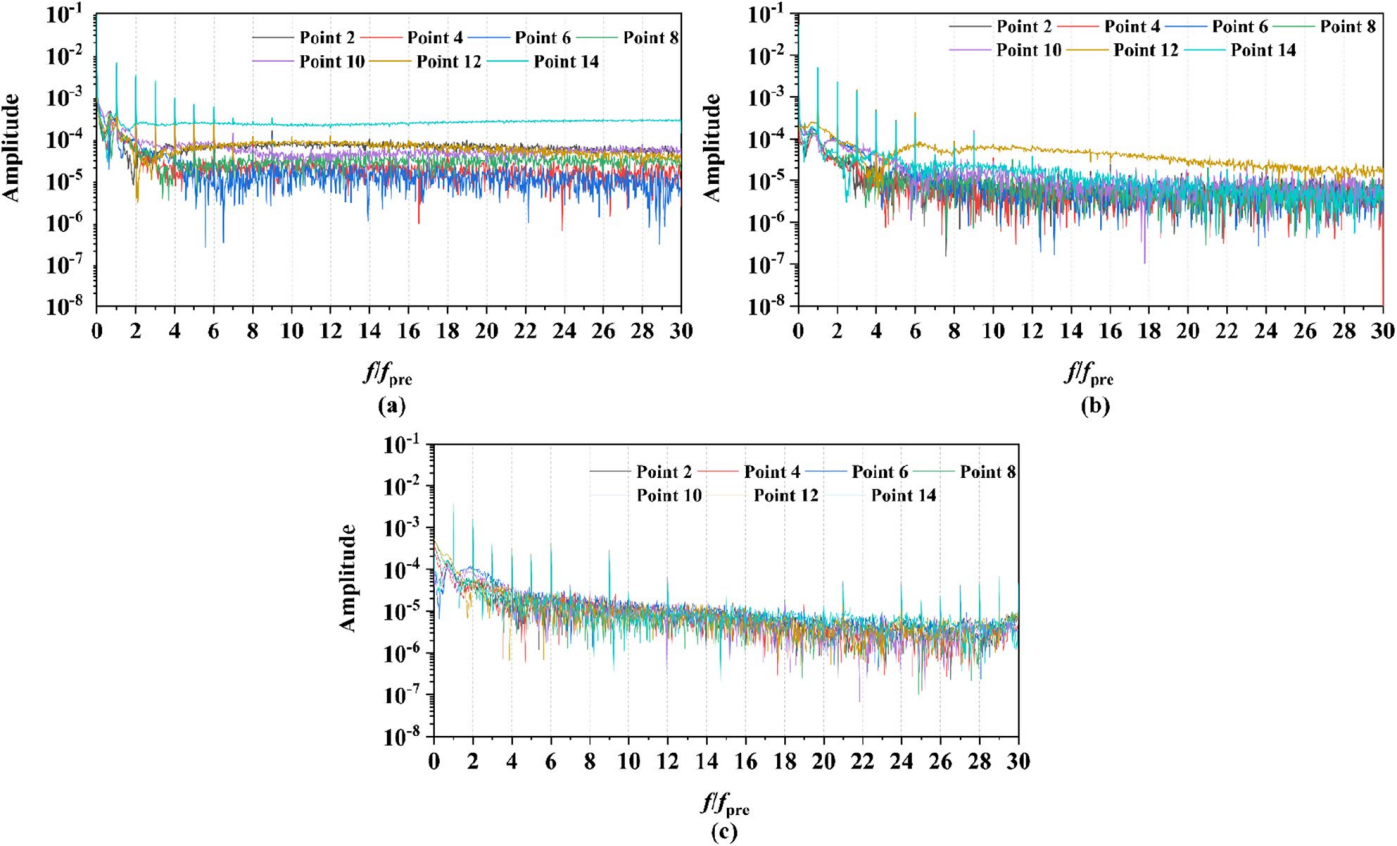
The frequency spectrum characteristics of Group 2 monitoring points in one period at the tip-clearance region of pump-jets with different f (from (**a**) to (**e**): *α* = − 4°, *α* = 0° and *α* = 4°.)

Two sets of monitor points between the rotor blade tip and inner surface are shown in Fig. 6, and the spatial distribution properties of pressure fluctuation of pump-jet with accelerating and decelerating ducts are shown in Figs. 23, 24, 25, 26, 27, 28, 29 and 30. Group 1 (P1, P3,…, P13) is set at the mid point of rotor blade tip, named by odd numbers. Group 2 is located in the passage between blades, there are corresponding points (P2, P4,…, P14) named by even numbers. The time history in one period and frequency amplitude of pressure fluctuation on Group 1 monitoring points of pump-jet with accelerating and decelerating ducts distinguished by *f* and *α* are shown in Figs. 23, 24, 27 and 28, respectively, while the pressure properties on Group 2 are exhibited in Figs. 25, 26, 29 and 30. The *Cp* of the monitoring points is periodically influenced by the rotor rotating as the peaks occur at the rotor blade passing frequency and the harmonic. In Figs. 23, 25, 27 and 29, the variations of the pressure fluctuation on the monitoring points Group 1 and Group 2 have a period of 1/3 T (where T is the rotation period of the hub: 1/40 s = 0.025 s). The time domain curves of pressure coefficient of monitoring points Group 1 own three peaks and three troughs relative to the quantity of pre-stator blade, induced by the pre-swirl influence on the rotor tip clearance. The *Cp* amplitude goes through an unstable wave from the troughs to the peak, caused by the tip-clearance flow, which carries low pressure appearing on the areas of monitor points. The frequency domain spectrum of monitoring points has the same peaks in *nf*_pre_.

The time domain and frequency domain curves of *Cp* fluctuation of pump-jet with accelerating and decelerating ducts distinguished by *f* are investigated in Figs. 23, 24, 25 and 26. In Fig. 23, the averaged value shows the upward tendency with the decreasing *f*, while the *Cp* curves share the smaller fluctuant range, except for *f* = 0.5t and − 0.5t. The wave between the troughs and the peak appears more intenser with the reduction of *f*, which indicated that the decreasing *f* leads to the more unstable tip-clearance flow. It is implied that *Cp* curves of rotor tip mid point on the pump-jet with decelerating ducts share the larger fluctuation, with the gentler tip-clearance flow. In the frequency domain curves in Fig. 24, except for *f* = − 0.5t, the reduction of amplitude at peaks and the averaged value is occurred by the decreasing *f*, which means that the decreasing *f* leads to the recline of tip clearance pressure distribution. Additionally, the difference between different points occurred more obviously, while the more obvious peaks appear at the high order of the harmonic, except for *f* = 0.5t and − 0.5t. Figures 25 and 26 shows the time domain and frequency domain curves of *Cp* fluctuation in one period on the monitoring points Group 2 which are located in the passage between rotor blades. In the time domain, the varied *f* virtually has no impact on the magnitude of pressure fluctuation, whereas the decreasing *f* results in the overall decline of pressure distribution. The tip-clearance flow through the passages between rotor blades presents a trough in *Cp* curves, with the large fluctuant range, implying that the relative low pressure appears in the rotor tip regions for most time of the whole rotational period. For the frequency domain curves, the amplitude at curves peaks is decayed more rapid as the camber becomes smaller, with the diminution of averaged value of amplitude. Compared with monitoring points in Group 1, *Cp* fluctuation curves of the monitoring points in Group 2 is smoother, resulting from the more complex tip-clearance flow in the tip region than that in the passages between rotor blades. The monitoring points in Group 2 share smaller magnitude fluctuation and more narrow range of peaks and broader range of troughs, while the troughs and peaks arrive further back in one period. Moreover, the averaged value of pressure coefficient on Group 2 of pump-je with decelerating duct performs higher than that on Group 1, while the pump-jet with accelerating duct presents opposite pattern. For the frequency domain curves, both amplitude fluctuation of pump-jet with accelerating and decelerating ducts perform smaller and more stable.

In order to research the spatial distribution properties of *Cp* fluctuation of pump-jet with accelerating and decelerating ducts distinguished by *α*, Fig. 27, 28, 29 and 30 compares the time domain and frequency domain curves of monitoring points in Group 1 and Group 2 on the pump-jets with different *α*. From Figs. 27 and 28, it is found that increasing *α* leads to the slight enhancement of the pressure fluctuation on the monitoring points in Group 1 and the averaged value of *Cp*. With the increment of *α*, the range of curve troughs appears broader, and the wave between the troughs and the peak becomes more drastic. In the frequency amplitude of pressure fluctuation, the difference between each monitoring point in Group 1 presents smaller results from the increasing *α*, whereas the magnitude of harmonic becomes more intense. In Fig. 29, pressure fluctuation curves of monitoring points in Group 2 located in the passage between rotor blades show the overall enhancement of pressure coefficient as the *α* go upward. The decreasing *α* improves the magnitude of pressure fluctuation, indicating that the tip-clearance flow of the pump-jet with decelerating duct is more stable, with less energy loss. In addition, the difference between the frequency domain spectrum of *Cp* on different monitoring points in Group 2 shows the downward trend as the attack angle increases. Meanwhile, increasing *α* results in the smaller averaged value of frequency amplitude, with the improvement of the rate of peak amplitude decay. The comparison between monitoring points in Group 1 and Group 2 presents the similar curve pattern with the pump-jet with accelerating and decelerating ducts distinguished by *f*.

### Transient flow field

#### Pressure field

**Figure 31:**
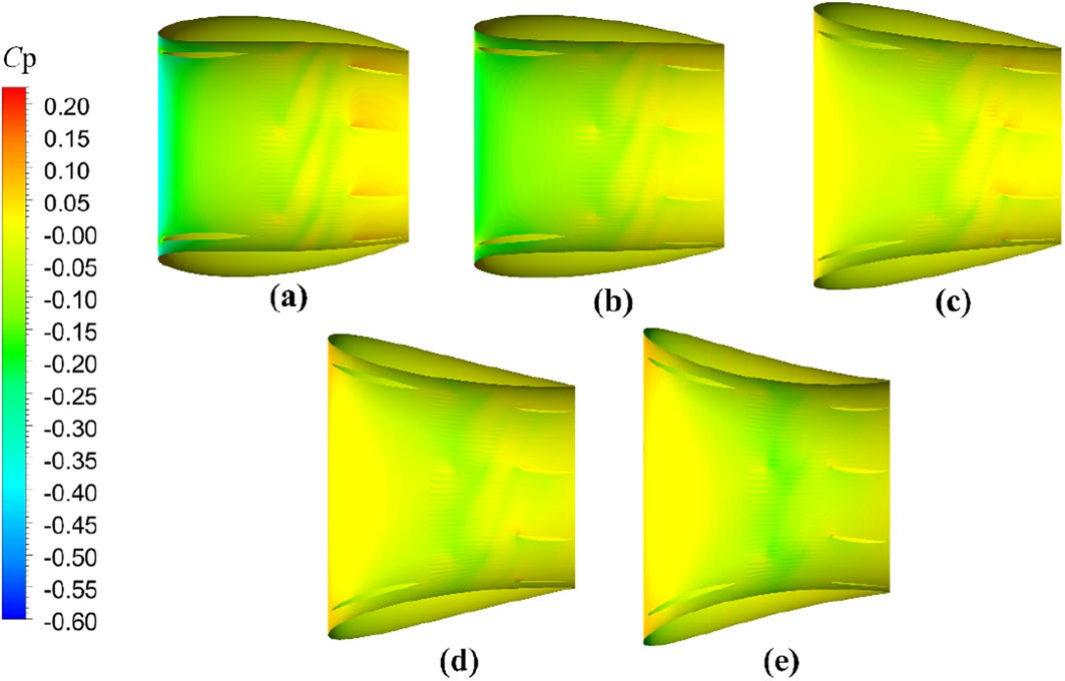
The pressure distribution on ducts as camber *f* = 0.5*t*, 0.25*t*, 0, − 0.25*t*, − 0.5*t.*

**Figure 32:**
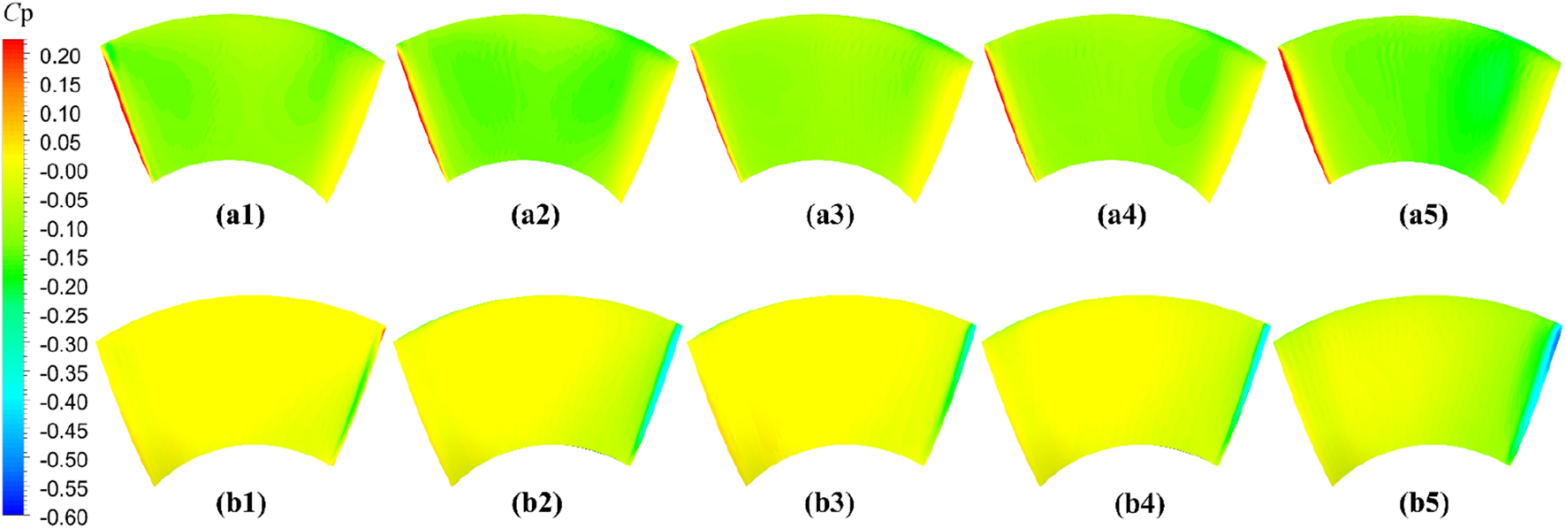
The pressure distribution on rotor blade as camber *f* = 0.5*t*, 0.25*t*, 0, − 0.25*t*, − 0.5*t.*

**Figure 33:**
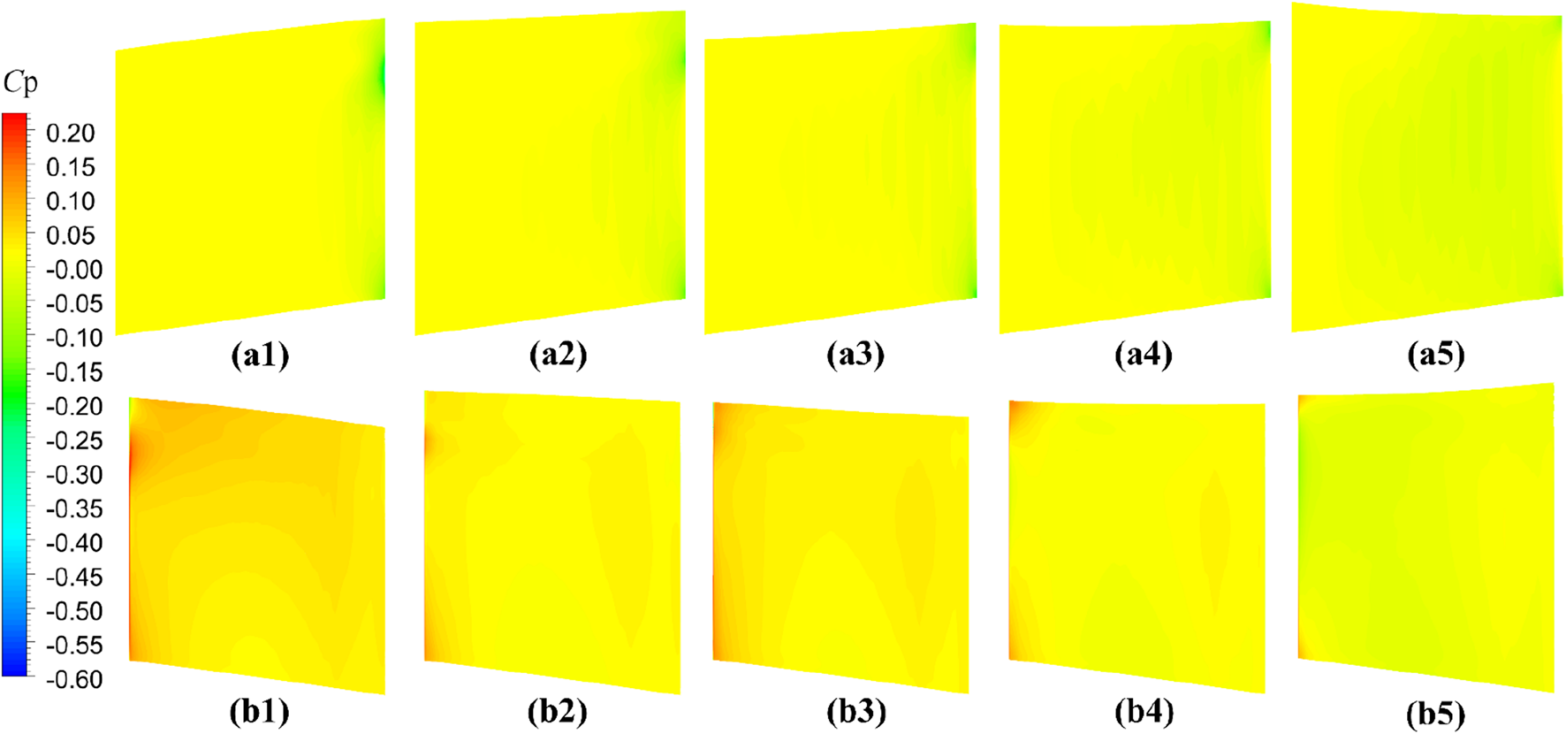
The pressure distribution on stator blade as camber *f* = 0.5*t*, 0.25*t*, 0, − 0.25*t*, − 0.5*t.*

**Figure 34:**
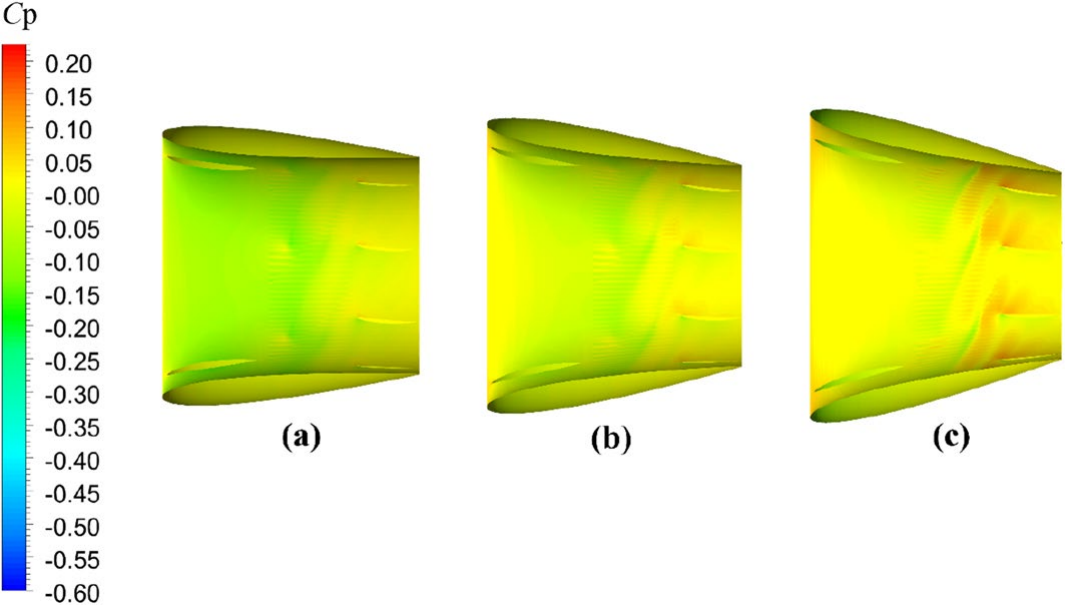
The pressure distribution on ducts as angles of attack *α* = − 4°, 0°, 4°

**Figure 35:**
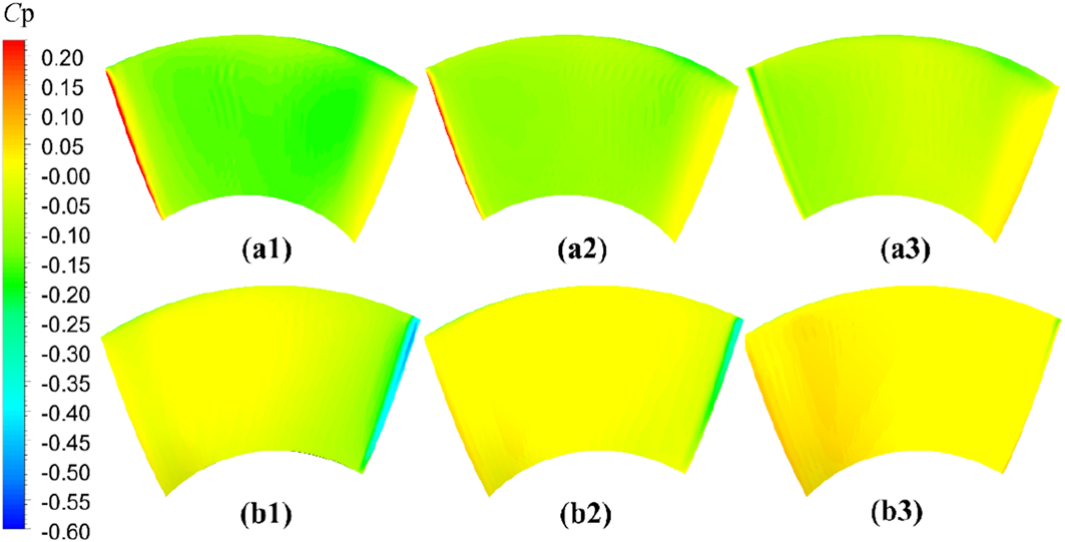
The pressure distribution on rotor blade as angles of attack *α* = 4°, 0°, − 4°

**Figure 36:**
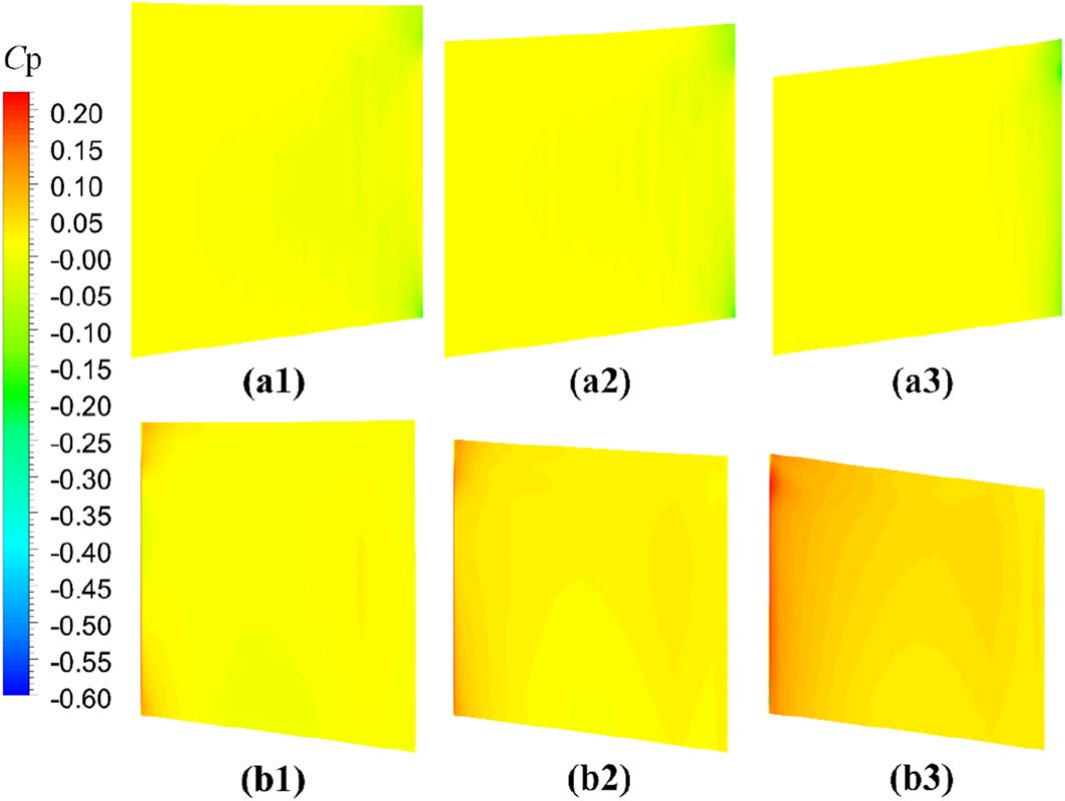
The pressure distribution on stator blade as angles of attack *α* = − 4°, 0°, 4°

The comparative evaluation of the open-water performance of pump-jets with accelerating and decelerating ducts, distinguished by *f* and *α* parameters, reveals that the pressure field exhibits characteristic properties due to the mutual interactions among the duct, stator, pre-stator, and rotor components. In the subsequent sections, the comparative effects of decelerating and accelerating ducts with varying *f* and *α* on pressure contours are discussed at a specific operating condition, *J* = 0.97 (*n* = 2400 r/min, *U* = 6 m/s). Figures 31, 32 and 33 depict the influence of *f* on the unsteady pressure contours on the duct, rotor, and stator, respectively, while Figs. 34, 35 and 36 illustrate the impacts of *α*. In Fig. 31, the pressure distribution on the exterior of the duct exhibits relative uniformity among ducts with different cambers. However, an increase in *f* not only results in a global rise in pressure on the exterior of the duct but also causes the high-pressure region to shift towards the leading edge inside the duct. Moreover, the low-pressure region on the exterior of the duct narrows with increasing *f* and the minimum pressure on the duct exterior coincides with the location of maximum curvature. Inside the duct, the pressure distribution is significantly affected by the rotor and stator regions. In the case of decelerating ducts (*f* = 0, 0.25*t*, 0.5*t*), the maximum high-pressure region primarily concentrates on the leading edge of the duct exterior and the inlet edge of the stator. Conversely, for accelerating ducts (*f* = − 0.25t and − 0.5t), the maximum high-pressure region is mainly concentrated on the leading edge of the duct exterior and the inlet edge of the stator. The tip clearance region and its downstream flow significantly influence the pressure distribution inside the duct, resulting in a noticeable low-pressure region near the rotor domain. Increasing *f* enhances the interaction between the tip clearance and the duct, leading to an increase in pressure in the high-pressure region located inside the duct near the rotor and stator, particularly on the pressure side of the stator region. This enhancement is advantageous for the cavitation resistance of the rotor and stator. Additionally, increasing *f* causes the low-pressure region at the inlet inside the duct to initially grow before decreasing. This indicates that the pressure in this region of decelerating ducts is lower than that of the accelerating ducts.

As depicted in Figs. 32 and 33, there is a notable intensity of pressure fluctuation at the leading edge of the rotor blade and the stator blade. On the suction side, two distinct low-pressure areas are observed, primarily located in the middle portion of the suction surface, one near the leading edge and the other near the trailing edge. Increasing the *f* parameter expands the low-pressure region near the leading edge and reduces its pressure value. Conversely, the lowest-pressure region near the trailing edge of the pump-jet with an accelerating duct, as influenced by *f*, is reduced in comparison to that of the accelerating duct without parameter variation. On the pressure side of the rotor blade, a significant high-pressure area occurs near the trailing edge. However, this high-pressure region in the pump-jet with an accelerating duct is smaller when compared to that with a decelerating duct. Decreasing *f* results in a leaning of the pressure on the rotor’s suction side, but the weakened interaction causes a reduction in pressure on the rotor pressure side, consequently leading to a loss of rotor thrust due to the larger pressure reduction on the pressure side. Furthermore, increasing *f* causes a global enhancement of pressure difference between the suction side and pressure side of the rotor blade, which is the primary factor affecting the trend of pump-jet thrust. This indicates that an accelerating duct, as influenced by *f*, increases the fluid velocity within the duct, thereby reducing the relative advance velocity of the rotor and resulting in a decrease in the thrust generated by the rotor. Conversely, a decelerating duct obtained by modifying *f* leads to the opposite trend in the flow field within the duct. The variation of *f* results in a significant change in the radial length of the stator blade and, consequently, alters the high-pressure area on the pressure side. Increasing *f* narrows the low-pressure region on the suction side of the stator blade, indicating that cavitation on the suction side is more likely to occur with a reduction in *f*. This corresponds well with the pressure distribution on the rotor blade, as shown in Fig. 32. Larger camber values of *f* are associated with an increase in duct thrust, intensified fluctuations at the leading edge of the stator, and higher pressure on the stator’s pressure side. Moreover, larger *f* strengthens the interaction between the rotor wake and the leading edge of the stator, but exacerbates the interaction between the tip of the leading edge of the rotor blade and the stator wake. Combined with the information from Figs. 32 and 33, it is evident that a larger *f* is more favorable for stator cavitation but places the leading edge under more favorable cavitation conditions.

The significant effects on pressure distribution in each component of the pump-jet, including the duct, rotor, and stator, due to changes in angles of attack *α*, are presented in Figs. 34, 35 and 36, respectively. Unlike variations in camber *f*, changes in angles of attack lead to simpler trends because they do not alter the shape of the duct profile. The alterations in *α* have no impact on the shape of the duct profile, leading to different phenomena. *α* results in opposite changes in pressure distributions for pump-jets with accelerating and decelerating ducts. Larger *α* corresponds to a reduction in duct thrust and overall pump-jet thrust, but it leads to intensified inner tip clearance flow and an increase in rotor load. The reason is that *α* does not offset the pressure increase on the inside of the duct downstream of the rotor. On the exterior of the duct, increasing angles of attack *α* lead to an overall decrease in pressure distribution and coefficient of pressure *C*_p_ at the leading edge of the duct. It also expands the low-pressure region. Combined with Fig. 34, it can be concluded that accelerating ducts increase the likelihood of cavitation erosion on the outside surface of the duct compared to decelerating ducts. Within the duct, raising *α* results in a broader area of high-pressure distribution on the inside surface of the duct, particularly noticeable at the duct inlet and near the stator region. Meanwhile, a pressure drop occurs at the leading edge of the duct exterior surface, and it does not compensate for the pressure increase on the inside surface within the rotor and stator downstream. This is the primary reason for the significant reduction in duct thrust. Increasing *α* improves the low-pressure region on the suction side of the rotor blade, reducing the pressure at the leading edge of the blade and expanding the area of this low-pressure region. This broader low-pressure region and lower pressure are particularly significant near the tip region and have a more pronounced influence on tip clearance flow on the suction side. Pressure experiences significant growth on the pressure side of the rotor, especially at the trailing edge, indicating a stronger interaction between the duct and rotor. Under these conditions, the *C*_p_ on the pressure side of the rotor increases, with a larger increment than on the suction side, leading to improved rotor blade thrust. Furthermore, increasing *α* induces a global increment in pressure on the overall stator blade and expands the high-pressure region on the stator’s pressure side from the leading edge area to the entire pressure side of the stator. However, the pressure distribution at the leading edge of the stator suction side decreases, and the low-pressure region expands from the bottom to the tip. This suggests that decreasing *α* can be used to mitigate cavitation. Unlike camber, decreasing *α* results in a regular increase in stator blade length in the radial direction, sharply narrowing the high-pressure region on the pressure side.

#### Velocity field

**Figure 37:**
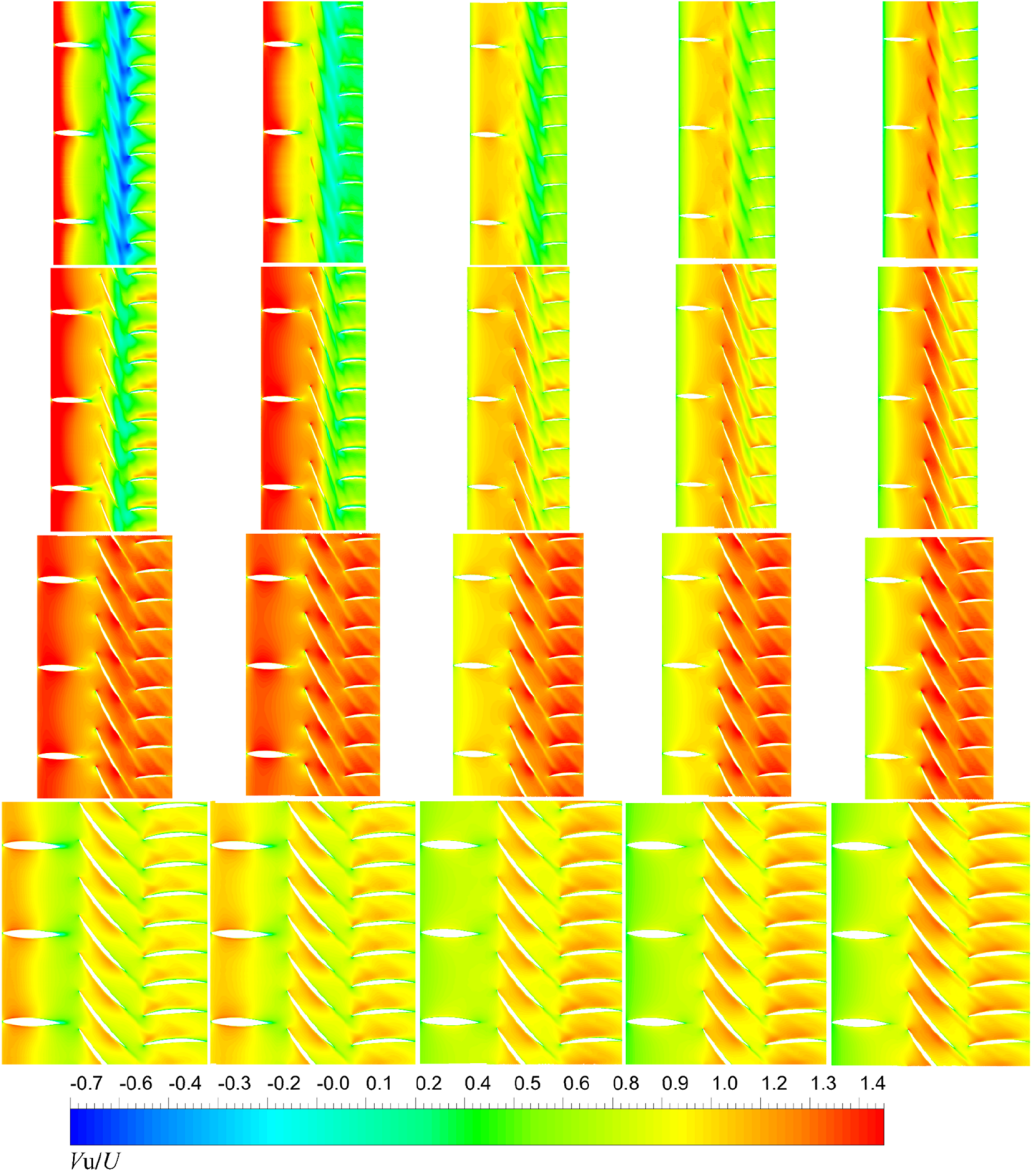
The velocity magnitude distribution at different radial places of pump-jets with different *f* (from left to right: *f* = 0.5t, *f* = 0.25t, *f* = 0, *f* = − 0.25t and *f* = − 0.5t. from top to bottom: span = 0.98, span = 0.9, span = 0.5, span = 0.02. span represents the relative location to the maximum distance between duct inner surface and hub.).

**Figure 38:**
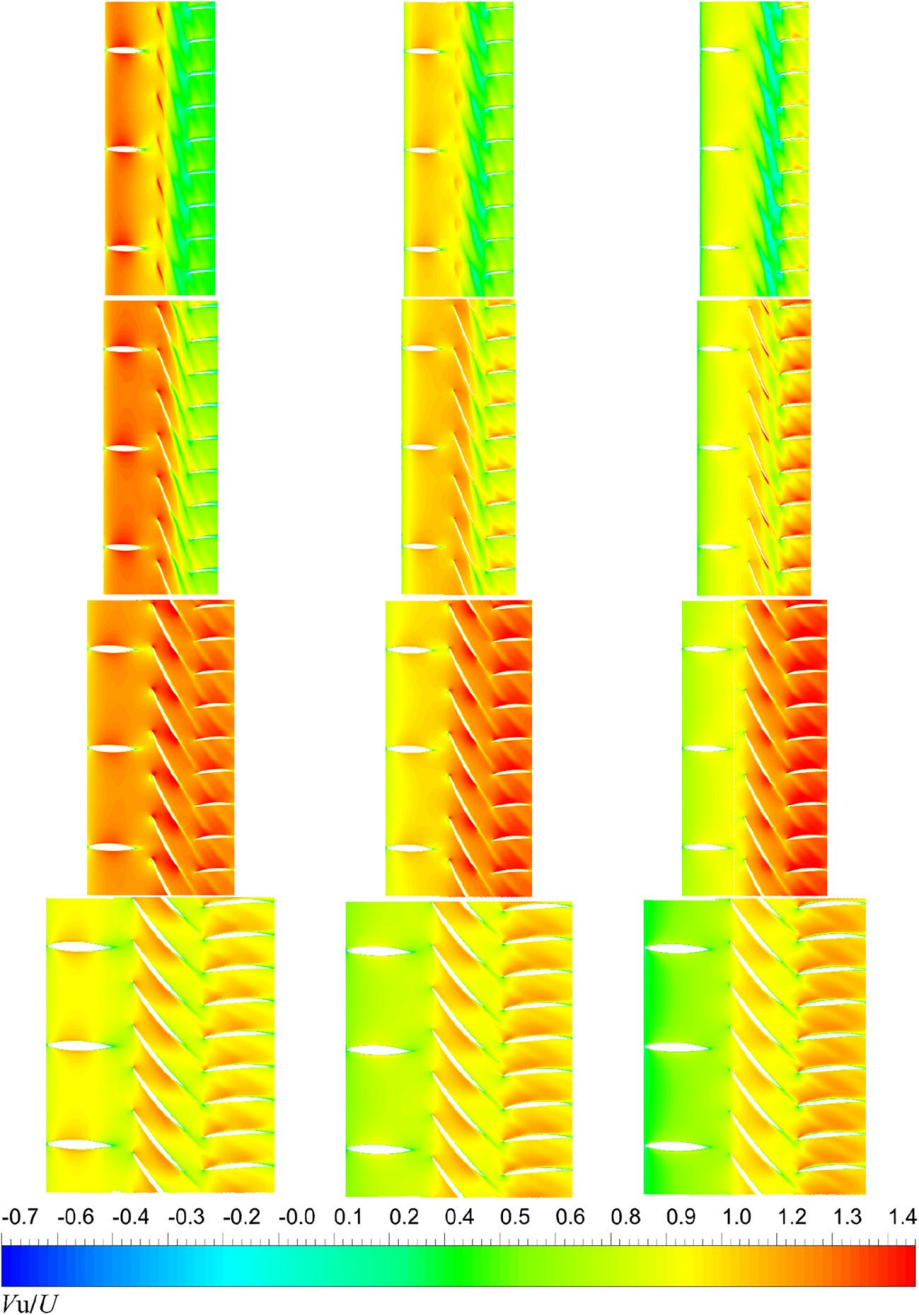
The velocity magnitude distribution at different radial places of pump-jets with different *α* (from left to right: *α* = − 4°, *α* = 0° and *α* = 4°. from top to bottom: span = 0.98, span = 0.9, span = 0.5, span = 0.02. span represents the relative location to the maximum distance between duct inner surface and hub.).

On the basis of the pressure distribution of unsteady results above, the impacts of accelerating and decelerating ducts on the pump-jet performance can be further concluded from the velocity field. With advance ratio *J* = 0.97 (*n* = 2400 RPM, *U* = 6 m/s). The axial velocity magnitude contours of the internal flow field at different radial places of transient simulations of pump-jets with accelerating and decelerating ducts distinguished by *f* and *α* are presented in Figs. 37 and 38. In Fig. 37, the axial velocity magnitude distribution of internal flow filed in the pump-jet with different *f*, from left to right presented as *f* = 0.5t, 0.25t, 0, − 0.25t and − 0.5t, while from top to bottom are represented as different radial locations from hub to tip region, correspond to *span* = 0.02, 0.5, 0.9 and 0.98, respectively. Overall, from hub to tip region, the averaged axial velocity distribution shows a downward trend after the increment at the middle of blade. It is found that the axial velocity of pump-jet with decelerating duct becomes smaller as the inflow passes through the pre-stator, while the higher-velocity region appears on the internal flow field of pump-jet with accelerating ducts as the inflow passes through the pre-stator. Increasing *f* leads to the improvement of the velocity distribution at the inlet area and the variation amplitude between the inlet and outlet of pump-jet, corresponding to the characteristics of accelerating and decelerating ducts. The low-velocity region occurs at the downstream area of rotor which is caused by the tip-clearance flow. Meanwhile, it becomes higher as the camber improves, indicating that increasing *f* results in the intenser unsteady tip-clearance flow. Additionally, the difference in axial velocity magnitude between different *f* becomes more apparent with the increment of radial places from span = 0.02 to span = 0.98. Compared with the contours of pump-jet accelerating and decelerating ducts modified by *f*, the velocity magnitude contours of pump-jet with different *α* show the similar overall pattern of accelerating and decelerating ducts in Fig. 38. Meanwhile, the velocity field of the pump-jet with accelerating and decelerating ducts obtained by varying *α* shares relatively more pronounced differences. However, the velocity distribution on the leading edge of rotor is induced by the increment of *α*. Moreover, the rising *α* significantly enhances the influence of tip-clearance, leading to the reduction of velocity distribution of the low-velocity region at the rotor trailing edge caused by the regular variation of the stator blade length in the radial direction.

**Figure 39:**
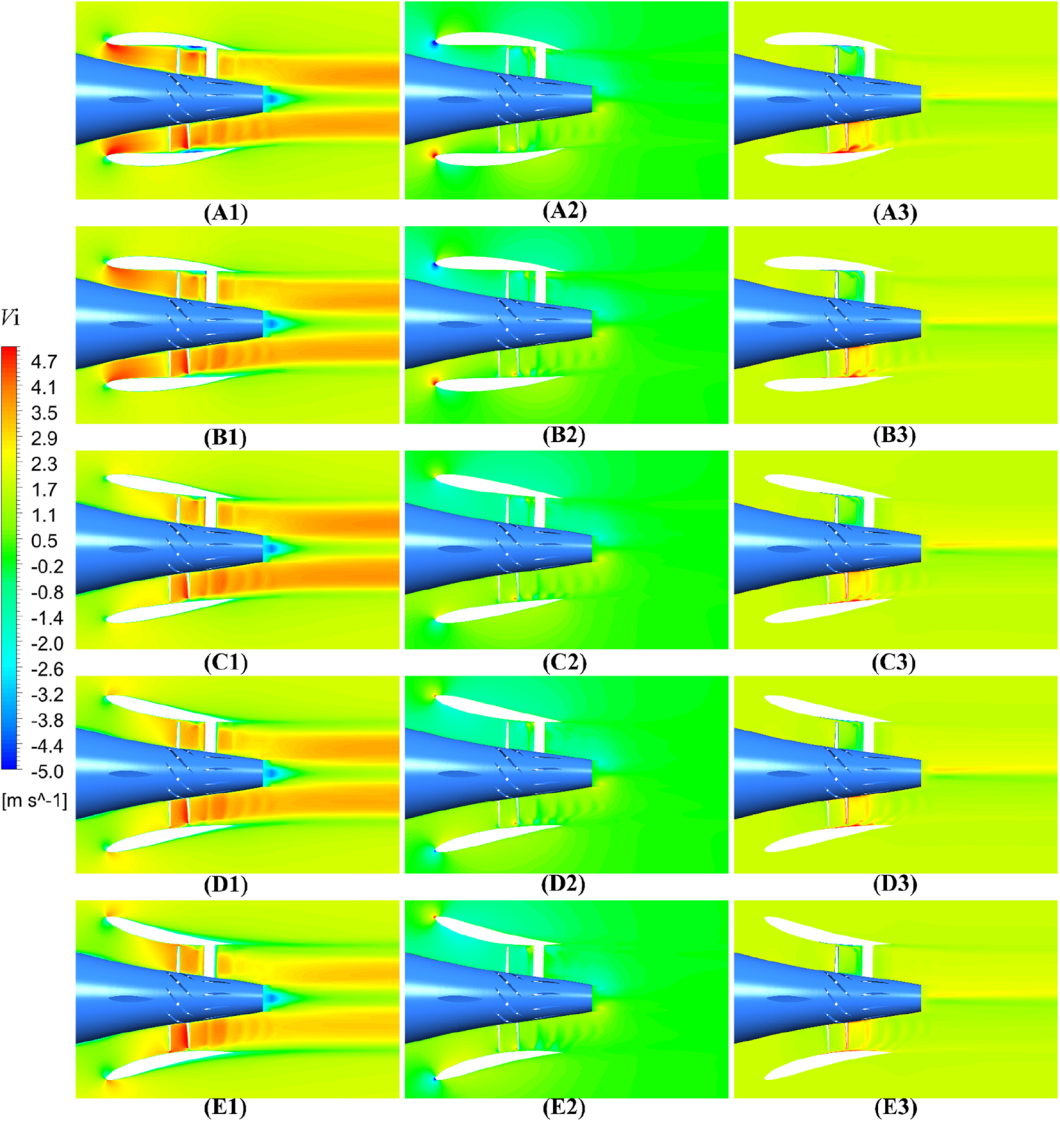
The velocity distribution of the x–y cut plane at different *f* (from (**A**) to (**E**): *f* = 0.5t, *f* = 0.25t, *f* = 0, *f* = − 0.25t and *f* = − 0.5t. from subscript 1 to subscript 3: axial velocity, circumferential velocity, radial velocity).

**Figure 40:**
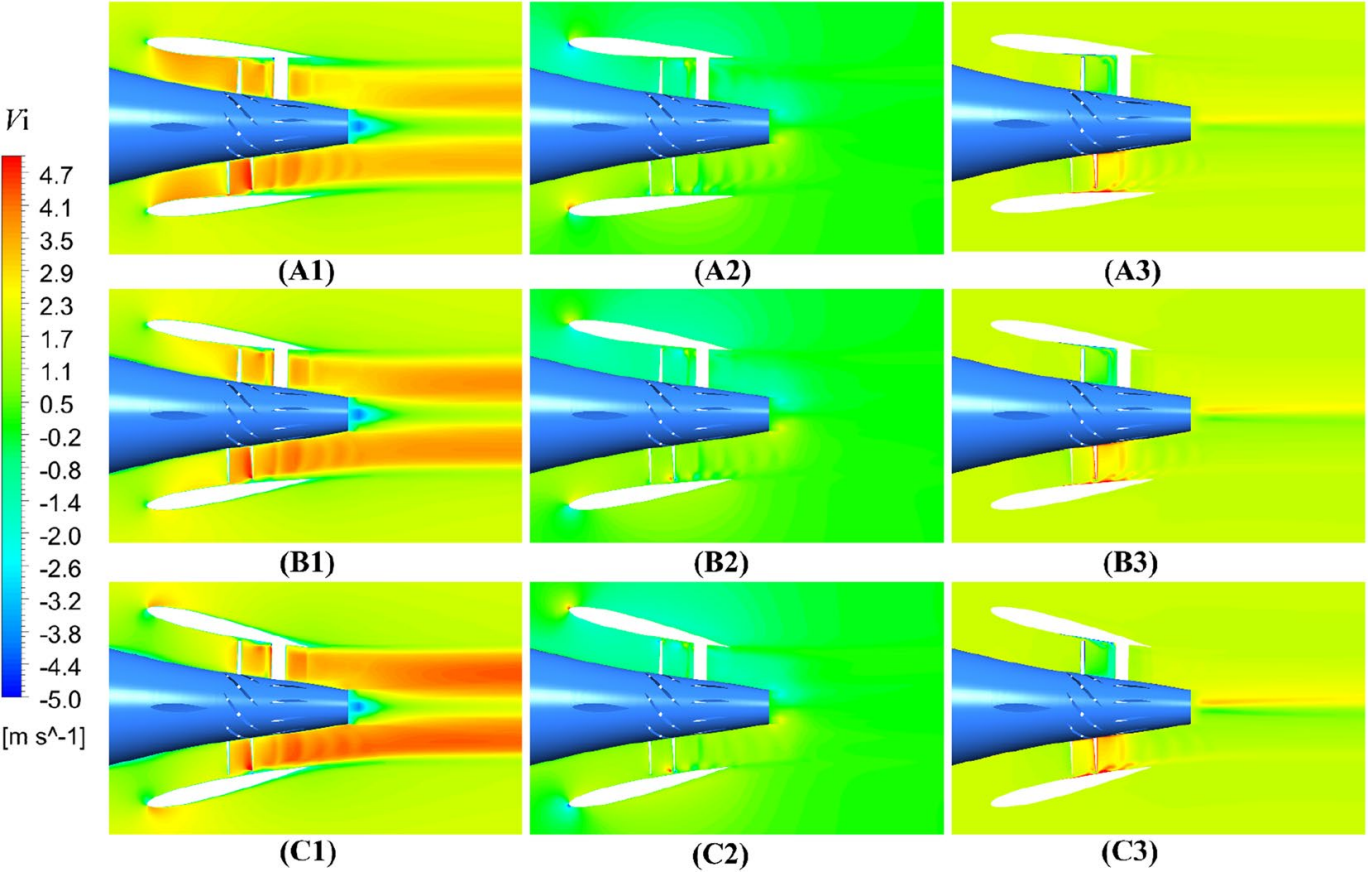
The velocity distribution of the x–y cut plane at different f (from (**A**) to (**E**): *α* = − 4°, *α* = 0° and *α* = 4°. from subscript 1 to subscript 3: axial velocity, circumferential velocity, radial velocity).

In Figs. 39 and 40, the velocity contours of pump-jet with different *f* and *α* on the axial velocity, the circumference velocity and radial velocity components in x–y plane are observed. Primarily, the axial component of velocity distribution is the dominant velocity constituent in the wake of the pump-jet. This axial velocity is generated by the acceleration of the rotor and the recycling effect induced by the stator. Meanwhile, the circumferential and radial velocity components are mainly produced by the rotational and acceleration effects of the rotor. The low-velocity region in the axial velocity component is most noticeable at the tip region downstream of the rotor, resulting from the generation of tip-clearance flow. It is worth noting that this low-velocity region in the axial velocity component, including the hub wake and tip region, exhibits high velocity values in the circumferential and radial components of the velocity distribution. Furthermore, the radial component of velocity distribution shows low velocity values, and in some cases, negative values. This is due to the internal flow field shrinking as a result of the angle between the hub and duct. The axial velocity distribution aligns with the results indicated in the velocity magnitude distribution at various radial positions in Figs. 38 and 39.

As shown in Fig. 39, the rising *f* gives rise to the increment of the pump-jet inlet velocity, owing to the movement of the locations of maximum curvature. In the axial component of velocity distribution, the low-velocity region generated by the tip-clearance flow shares lower value as the camber increases, correspondingly presenting the higher velocity distribution in the circumferential distribution. In the radial components of velocity, the velocity distribution is slightly influenced by the variation of *f*, owing to the slight change in the shrinking extent of the internal flow field generated by the angle between the hub and duct. With the reduction of *f*, the outlet velocity in axial direction gradually drops off, caused by the higher energy loss. The radial velocity distribution at the leading edge of duct shares the opposite pattern between accelerating and decelerating ducts. In Fig. 40, it is observed that the area of low-velocity region narrows as the attack angle increases, with the improvement of the axial velocity in the wake of pump-jet. The radial velocity distribution at the leading edge of duct shares the similar pattern with the accelerating and decelerating ducts varied by *f*. In addition, the increasing *α* leads to the higher axial velocity of pump-jet outlet, indicating that the acceleration effect of accelerating duct modified by *α* is more prominent than that modified by *f* (Fig. 40).

**Figure 41:**
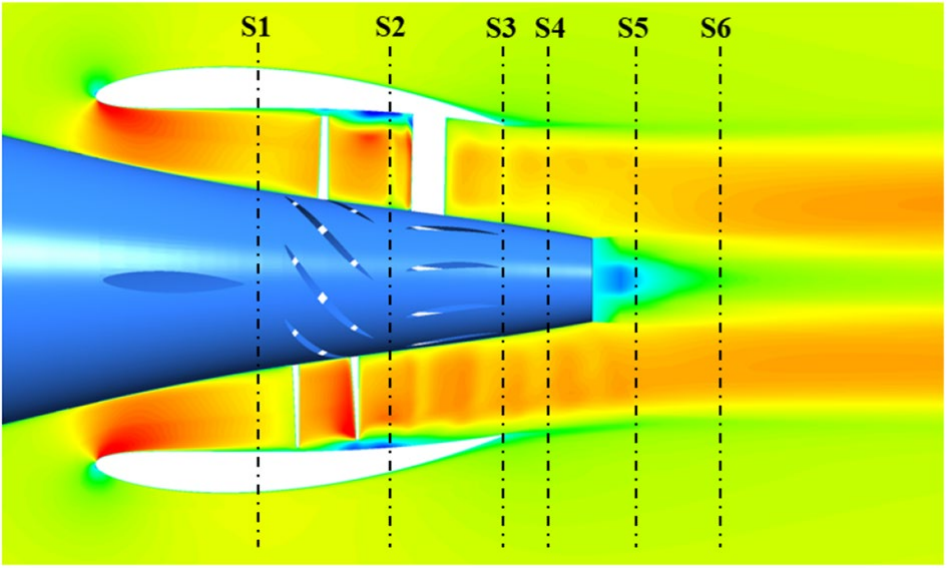
The schematic of the positions of selected slices (from S1 to S6: location between pre-stator and rotor, location between rotor and stator, the outlet of pump-jet, 3/4*D*r, 4/4*D*r and 5/4*D*r).

#### Vorticity distribution

**Figure 42:**
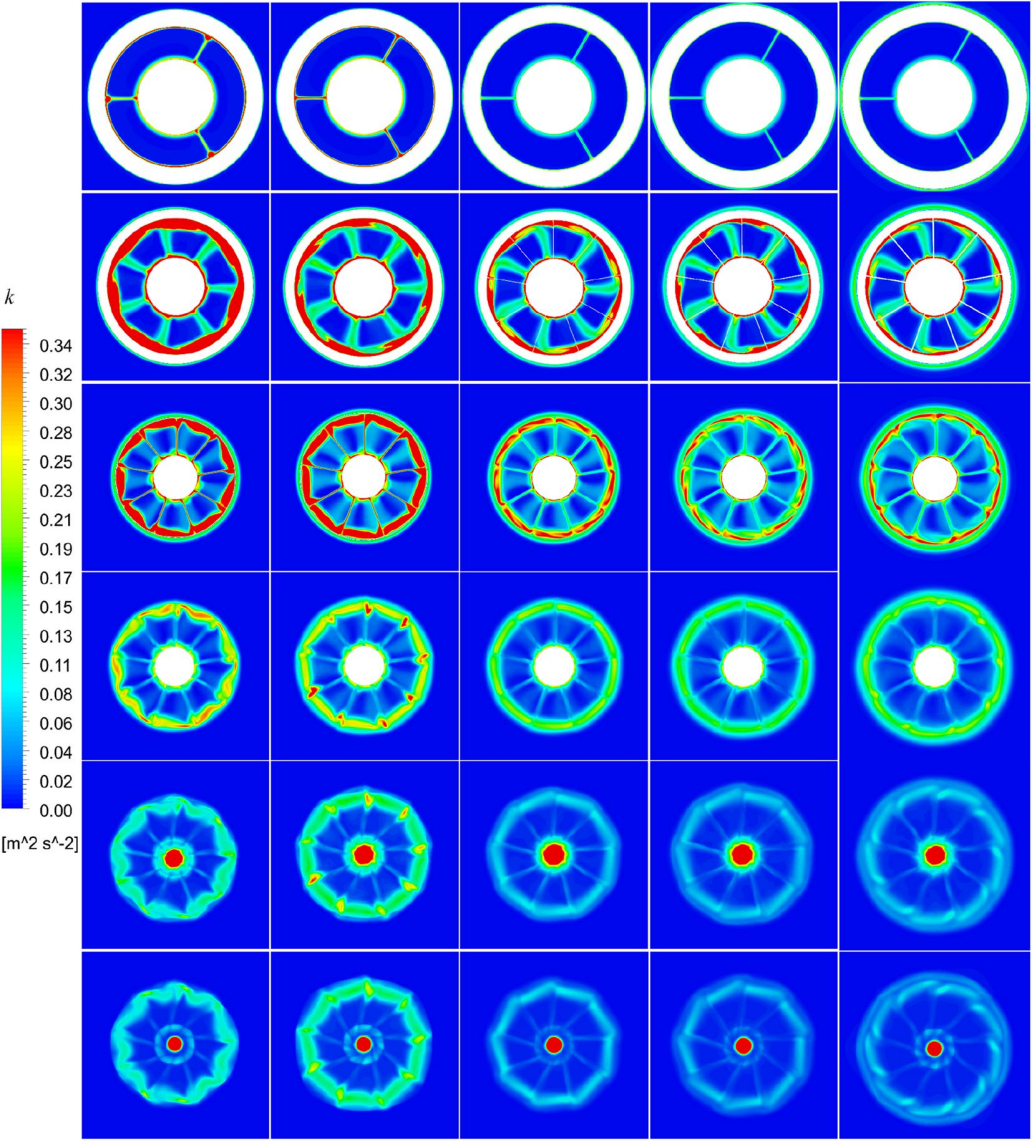
The turbulence kinetic energy distribution at different slices of pump-jet with different *f* (from top to bottom: S1 to S6. from left to right: *f* = 0.5t, *f* = 0.25t, *f* = 0, *f* = − 0.25t and *f* = − 0.5t.).

**Figure 43:**
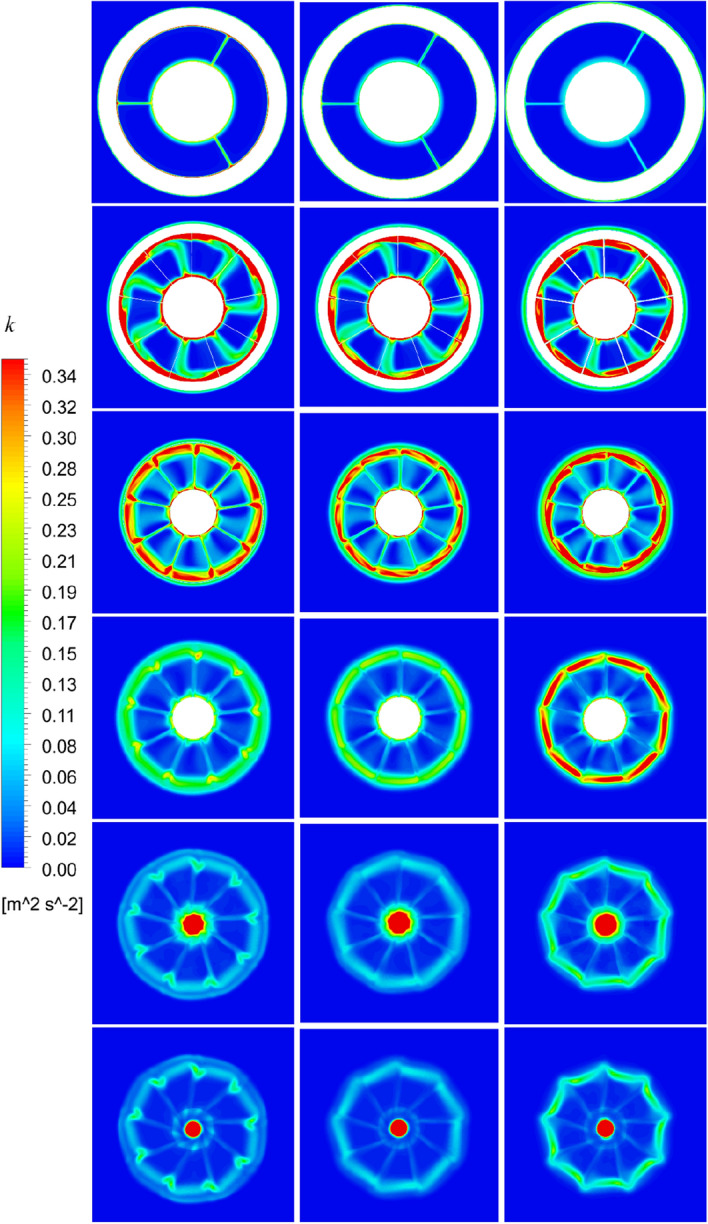
The turbulence kinetic energy distribution at different slices of pump-jet with different f (from top to bottom: S1 to S6. from left to right: *α* = − 4°, *α* = 0° and *α* = 4°).

**Figure 44:**
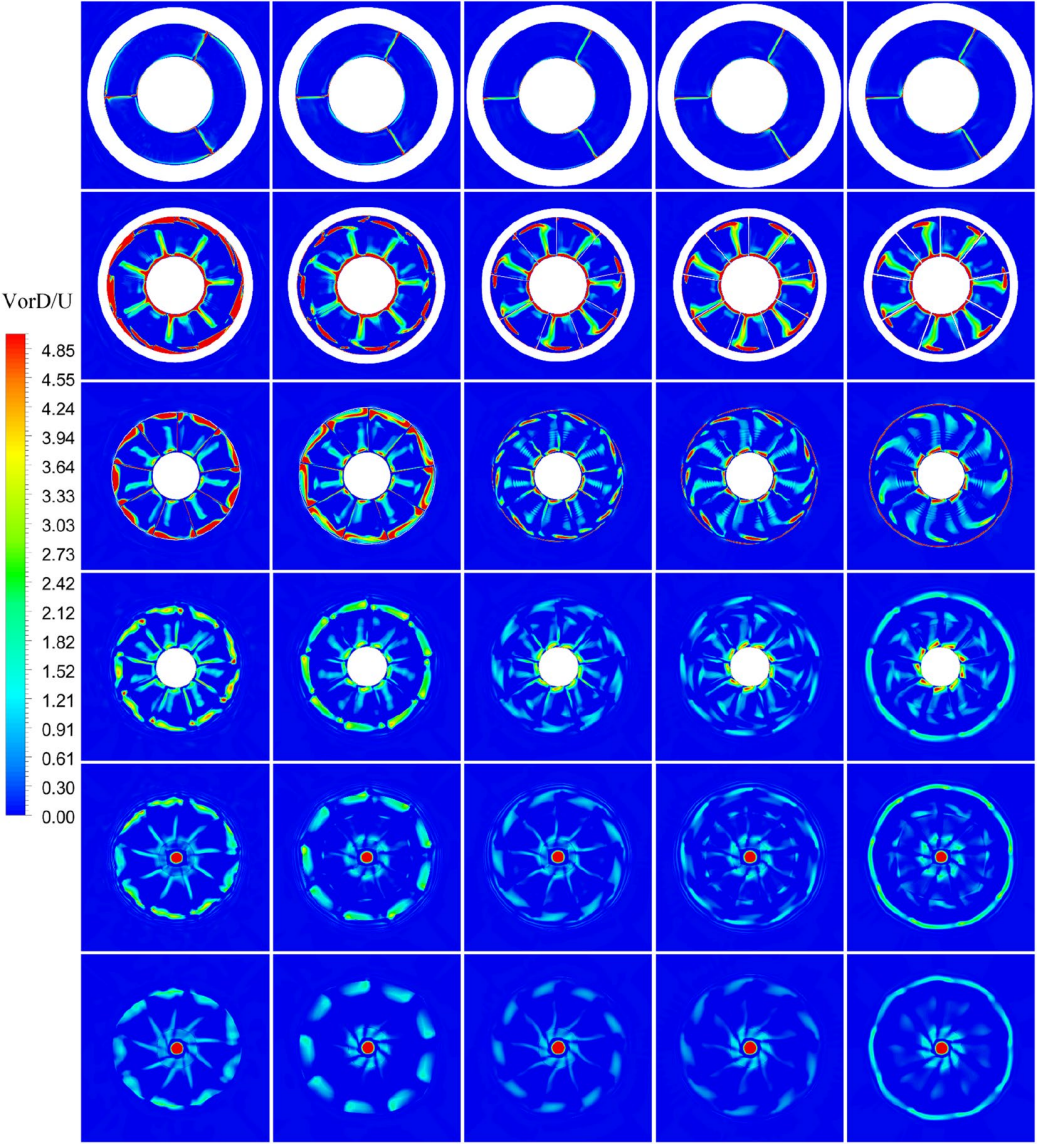
Views of the vortices of PJP: the vorticity magnitude of the pump-jet with different *f* in the x–y plane normalized with U/D (from top to bottom: S1 to S6. from left to right: *f* = 0.5t, *f* = 0.25t, *f* = 0, *f* = − 0.25t and *f* = − 0.5t).

**Figure 45:**
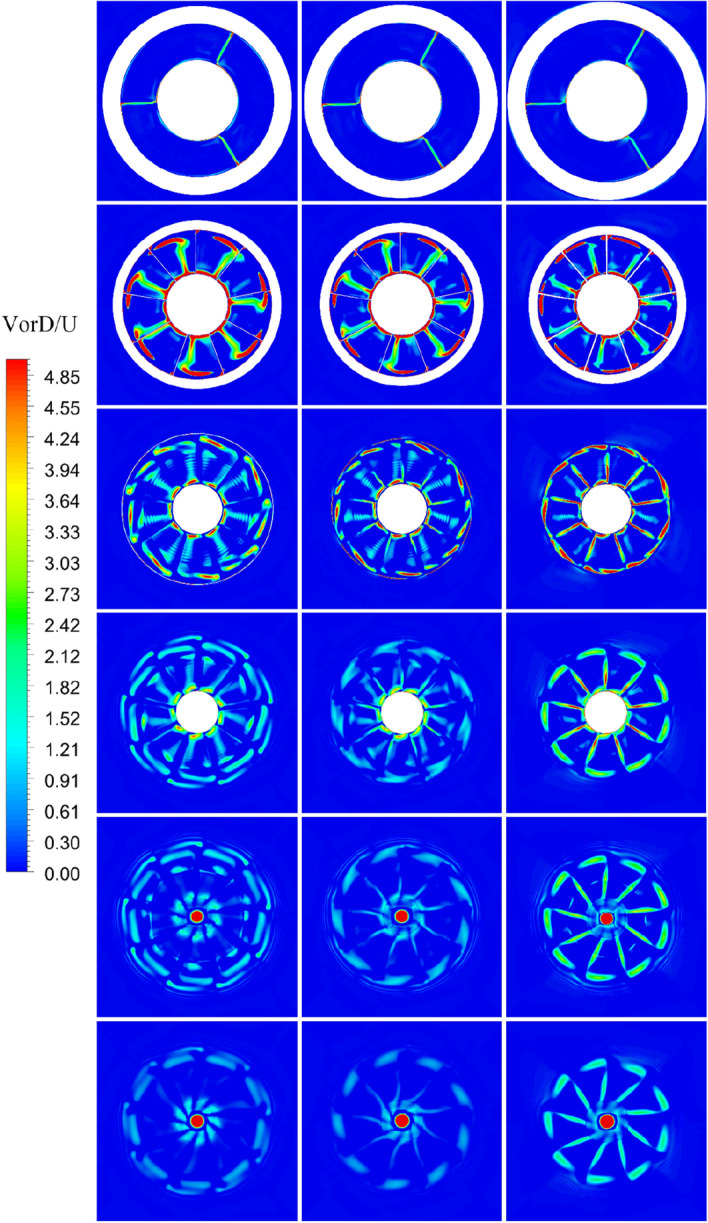
Views of the vortices of PJP: the vorticity magnitude of the pump-jet with different *f* in the x–y plane normalized with U/D (from top to bottom: S1 to S6. from left to right: α = − 4°, α = 0° and α = 4°).

The turbulence kinetic energy *k* and the vorticity magnitude distribution *VorD*/*U* at different x positions of pump-jet with accelerating and decelerating ducts distinguished by *f* and *α* at different sections, including sections S1 to S6, are shown in Figs. 41, 42, 43 and 44. Taking the pump-jet with *f* = 0.5t as an example, the locations of the slices in these figures are selected as Fig. 45, while S1 to S6 represent the position between pre-stator and rotor, the position between rotor and stator, the outlet of pump-jet, 3/4*D*r, 4/4*D*r and 5/4*D*r, respectively. The selected slices in pump-jets with other duct profile parameters are the same as the position in Fig. 40.

On the whole, the positions in the tip region in rotor and stator domain and the central area after the pump-jet hub shares the high *k* level, induced by the occurrence of the rotor tip vortices and hub vortices instability. Combined with *k* distribution, it is found from the vorticity magnitude distribution that increasing x distance inside the pump-jet leads to the bigger angular displacement between the tip vortex and trailing vortex, resulting from the procedure of trailing edge vortices rolling up. Owing to the straight blade construction of the pre-stator, the intensity of pre-stator trailing vortices is much lower than that of the rotor and stator blades, while the pre-stator trailing root vortices occur in the slice between pre-stator and rotor. From position S2, the rotor trailing vortex shares the intense mutual interaction with the tip leakage vortex, due to the gradual contact of the tip region of the rotor wake and the tip leakage vortices. It is observed by slices S2 and S3, the rotor trailing vortex, shedding from 7 blades, is interacted with the pre-stator trailing vortex, while the mutual interaction is appeared between the hub vortex and the trailing vortex which is generated by the shedding of 9 blades. From the concern of S4 to S6, the apparent stator trailing root vortices, stator trailing edge vortices and hub vortices instability are occurred in the wake of pump-jet.

In Figs. 42 and 44, it is found that decreasing *f* leads to the higher intensity of vortices generated by the pump-jet. Specially, the tip-clearance vortices are stronger in the pump-jet with decelerating duct, while the outlet of pump-jet with accelerating duct shares the stronger rolling up process. Moreover, the more unstable mutual interaction between the rotor trailing awake and the stator trailing vortices appears with the decline *f*. The slices in the wake of pump-jet demonstrate that the reduction of *f* also enhance the stator trailing root vortices, while the rotor trailing wake flows through the stator blades and becomes more uniform, especially pronounced at *f* = 0.25t. Except for *f* = 0.5t and − 0.5t, the decline of intensity of duct-induced vortex is occurred along with the decreasing *f*. As shown in Figs. 43 and 45, the variation of *α* shows the similar pattern with the accelerating and decelerating ducts by the change of *f*, while the significant differences of vorticity distribution are also appeared in slices. In S2, the rotor trailing vortices shares stronger intensity as the *α* improves. In the wake of pump-jet, increasing *α* enhances the intensity of trailing vortices, whereas the vortex distribution occurs more un-uniform caused by the stronger rotor trailing vortices. Additionally, the bigger *α* results in the stronger rolling up process, with the less unstable mutual interaction between the rotor trailing awake and the stator trailing vortices.

**Figure 46:**
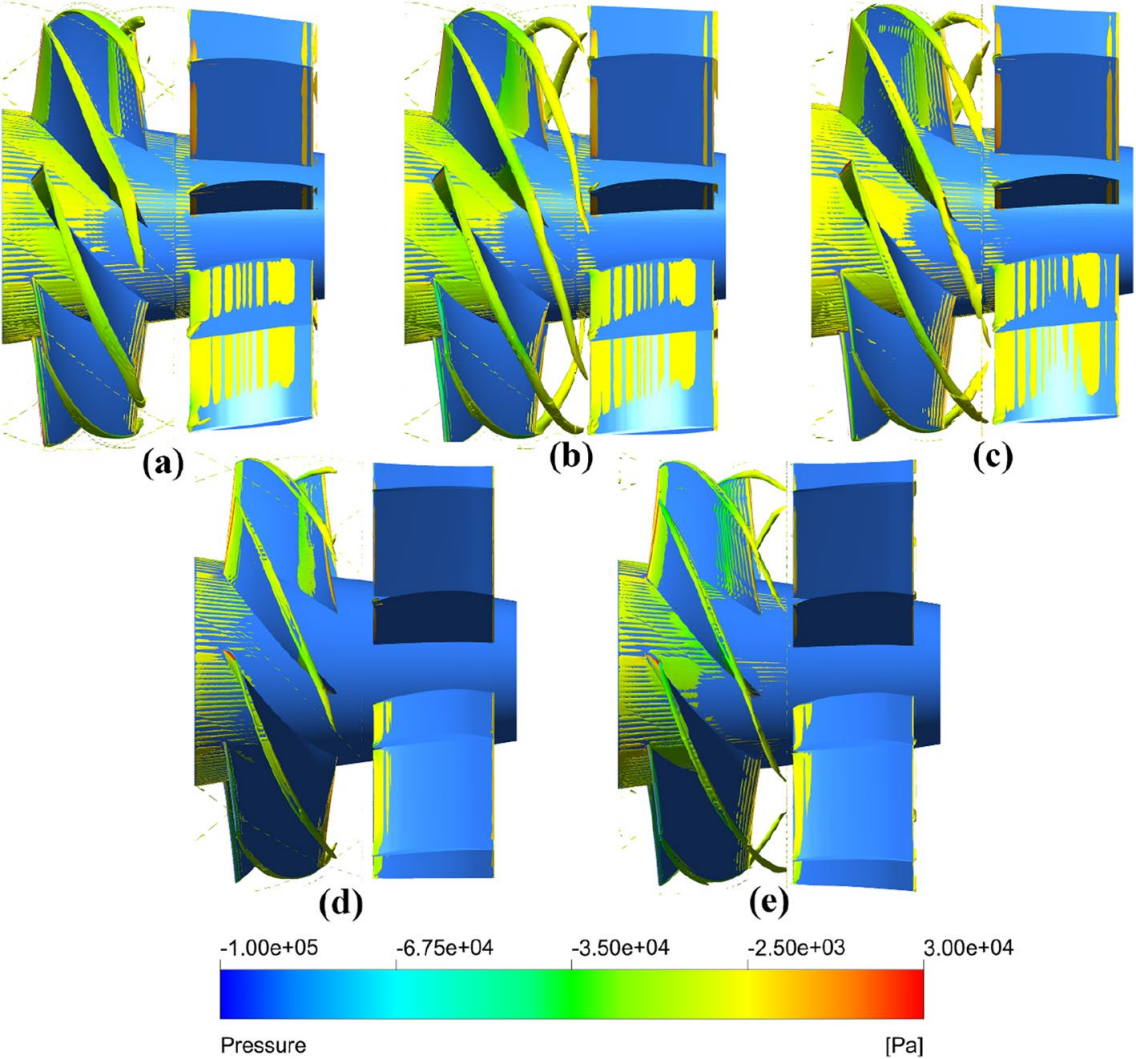
The vortices iso-surface with *Q* = 1 × 10^6^ s^−2^ inside the pump-jet with different *f* colored with pressure (from (**a**) to (**e**): *f* = 0.5t, *f* = 0.25t, *f* = 0, *f* = − 0.25t and *f* = − 0.5t).

**Figure 47:**
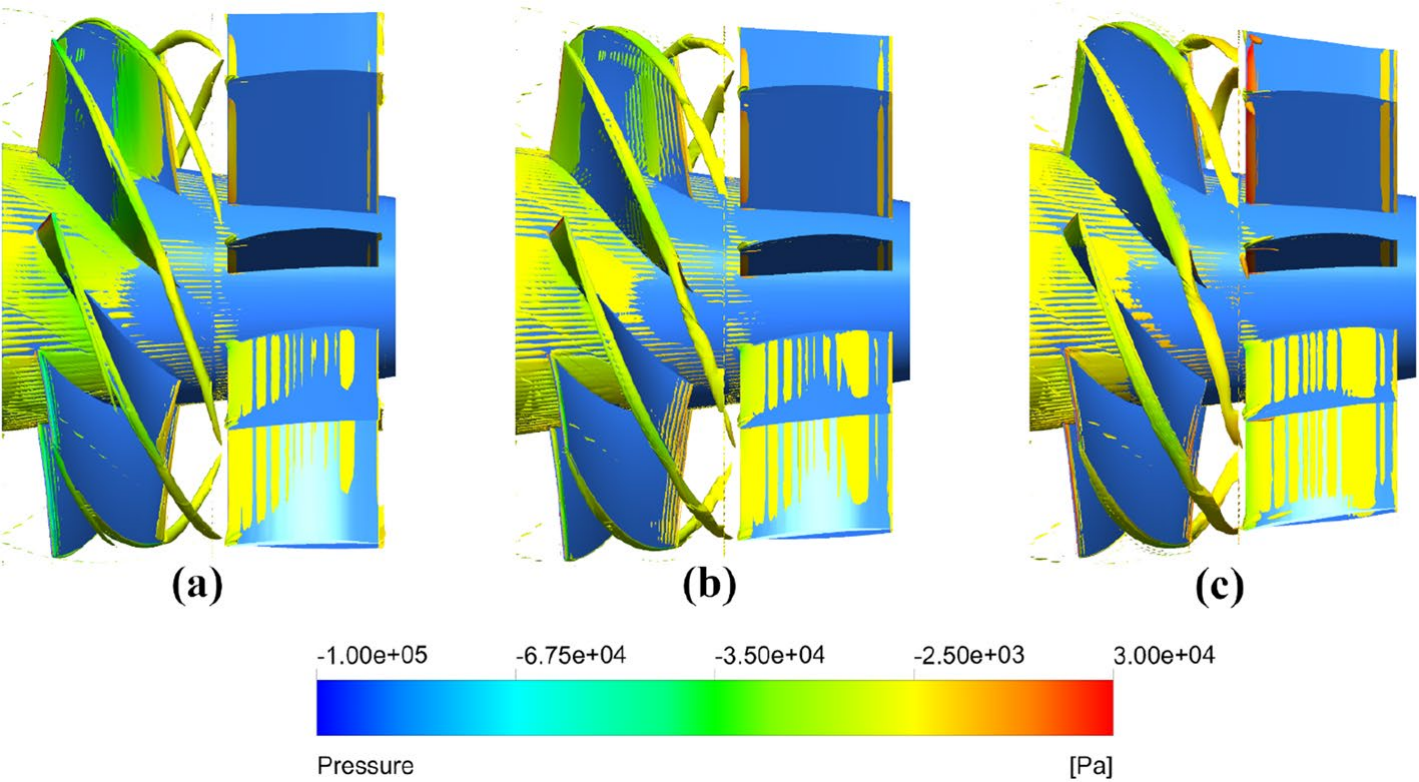
The vortices iso-surface with *Q* = 1 × 10^6^ s^−2^ inside the pump-jet with different *α* colored with pressure (from (**a**) to (**c**): *α* = − 4°, *α* = 0° and *α* = 4°).

In order to further investigate the vorticity distribution of pump-jet with accelerating and decelerating duct varied by *f* and *α*, the instantaneous iso-surfaces of vorticity magnitude are demonstrated in Figs. 46 and 47. The variation in the intensity of vortices is mainly caused by the tip-clearance flow change induced by the *f* and *α*. The tip vortices are apparently generated in the tip region between the rotor and duct, which starts at the leading edge of the rotor blade tip and extends backward in a slender strip with the direction of rotor rotation. Meanwhile, the obvious vorticity distribution occurs at the locations on the leading edge, trailing edge, and suction side. It is found that the stators in all of the pump-jets shares the relative well effects of recovering tip-clearance flow, benefiting to the fluctuation and stability of flow field. In Fig. 46, the angle between the vortex line and the rotor blade gradually improves along with the decline of *f*, as well as the increment of the intensity of tip vortices. The instability of tip vortices becomes intenser in the pump-jet with accelerating duct, even with bifurcation. Meanwhile, the area of the vortices occupied on the suction side of the stator narrows, whereas the length of the tip vortices becomes longer, caused by the stronger effect of radial velocity. It corresponds to the pattern indicated in the vorticity magnitude in Figs. 42 and 44. It is concluded that the rotor trailing vortices have significant effects on the flow field, while it becomes stronger by decreasing *f*. As shown in Fig. 47, the results are also in accord with the vorticity magnitude exhibited in Figs. 43 and 45. It is observed from Fig. 47 that the increasing *α* has a slight impact on the length of tip vortices, but leads to the enhancement of rotor trailing vortices. The occupation of the vorticity distribution on the suction side of stator blade also expand to mostly whole suction side surface from *α* = − 4° to *α* = 4°. Additionally, the angle between the vortex line and the rotor blade gradually improves along with the increment of *α*, as well as the increment of the unevenness of tip vortices. Similar with the tip vortices in the pump-jet with different f, the instability of tip vortices also becomes higher in the pump-jet with accelerating duct.

## Conclusion

In this study, the exploration of the impacts posed by accelerating and decelerating ducts with distinct parameters of *f* and *α* on the transient flow field is carried out using a combination of numerical analysis techniques. Specifically, the Reynolds-averaged Navier–Stokes equations (RANSE) method and Detached Eddy Simulation (DES) model, complemented by sliding mesh technology, are employed to thoroughly examine the flow characteristics and interactions of the pump-jet with varying duct profile parameters. To scrutinize the intricate effects of different duct profiles, the study considers pump-jets with distinct camber sizes (*f* = 0.5t, 0.25t, 0, − 0.25t, − 0.5t) and angles of attack (*α* = 4°, 0°, − 4°). The investigation is structured around two main focal points: first, a comprehensive comparison of the open water performance of pump-jets equipped with two types of ducts differentiated by varying *f* and *α* values; second, a detailed examination of the transient flow characteristics exhibited by these pump-jets. The analytical efforts culminate in the synthesis of primary results and conclusions that shed light on the intricate interplay between pump-jet performance and duct profile parameters.

- Concerning the overall pump-jet performance curves, augmenting the parameter *f* results in a sharper ascent of the propulsion efficiency until it reaches its apex. The similarity between the performance curve trends in terms of *α* and *f* arises from the fact that alterations in *α* do not influence the shape of the duct profile. Consequently, the resulting trends become more straightforward and pronounced. However, when it comes to the parameter *f*, while an increase does improve propulsion performance, surpassing a certain threshold can lead to significant performance degradation, especially at lower *J* values. In a comprehensive assessment, it is evident that the decelerating duct is adept at sustaining high hydrodynamic efficiency over a wider range of *J* values in comparison to the accelerating ducts. This observation highlights the beneficial impact of utilizing decelerating duct configurations in terms of maintaining propulsion efficiency across different operational conditions.
- The mutual interference among the rotor, duct and stator results in the exciting force of the rotor and stator blades. Time domain curves showed periodic fluctuations, related to the blade number and the rotational speed of rotor. Decreasing *f* not only reduces the average value of unsteady rotor thrust, but also lowers the differences in rotor thrust coefficient values between the peak and trough of curves. The fluctuation magnitude of stator shows an upward trend with the increment of *f*, except for *f* = 0.5t and − 0.5t. Increasing *α* improves average unsteady force of rotor and stator, whereas the opposite pattern appears in the magnitude of rotor and stator. For the frequency domain analysis, increasing *f* results in the enhancement of thrust and torque as the fluctuation and time-averaged values are improved, while the values of peaks are decayed more rapidly. Additionally, the minimum difference between different rotor blades occurs in *f* = 0.25t. Higher *α* leads to reduced amplitudes at peaks and averaged values, and the internal flow field between rotor and stator performs the enhanced instability.
- The spatial distribution properties of pressure fluctuation in pump-jets with accelerating and decelerating ducts are analyzed by monitor points surround the rotor blades. The monitoring points exhibit three peaks relative to the pre-stator blade quantity, influenced by pre-swirl and tip-clearance flow. Time and frequency domain analysis revealed periodic influences on pressure fluctuation, with peaks occurring at the blade passing frequency and harmonics. Both amplitude fluctuation of pump-jet with accelerating and decelerating ducts performs smaller and more stable. Meanwhile, it is indicated that the tip-clearance flow of the pump-jet with decelerating duct is more stable, with less energy loss. *Cp* curves of rotor tip mid point on the pump-jet with decelerating ducts share the larger fluctuation, with the gentler tip-clearance flow.
- The *f* and *α* significantly influences the pressure distribution on the whole components of pump-jet. Increasing *f* not only raises the pressure globally on the whole pump-jet, but also causes the expansion of high-pressure area. It results in the relatively higher hydrodynamic loading and decreases the likelihood of cavitation at the leading edge. Moreover, larger *f* strengthens the mutual effects between the rotor wake and the leading edge of stator, benefiting to the cavitation resistance of rotor and stator. However, it also intensifies the mutual impacts between the tip of leading edge of rotor blade and the stator wake. Change *α* has no effect on the duct profile which results in a relatively different outcome. Decreasing *α* narrows the high-pressure region on pressure side sharply, while increasing leads to more intense inner tip clearance flow and better rotor load, but reduces the pump-jet thrust. Decreasing *α* results in an increase in the likelihood of cavitation at the leading edge. The pump-jet with lower *α* generates bigger thrust.
- The analysis of the velocity field provides insights into the impact of accelerating and decelerating ducts on pump-jet performance. Pump-jets with decelerating ducts exhibit reduced axial velocity as the flow passes through the pre-stator, while pump-jets with accelerating ducts show higher velocity regions after the pre-stator. Increasing *f* improves the velocity distribution at the inlet and increases the variation between the inlet and outlet, reflecting the characteristics of accelerating and decelerating ducts. The tip-clearance flow causes a low-velocity region downstream of the rotor, which becomes more pronounced with higher *f* values. Comparisons between different *f* and *α* demonstrate similar patterns in velocity distribution, but variations in *α* have a more significant influence on the tip-clearance flow and velocity distribution at the rotor trailing edge. Overall, increasing *α* enhances the acceleration effect of accelerating ducts and leads to higher outlet axial velocity, while decreasing *f* results in reduced outlet velocity due to higher energy loss.
- The investigation of complicated vortex system in flow field is carried out by the analysis of *k* and vorticity magnitude distribution at different positions. Increasing distance inside the pump-jet leads to a larger angular displacement between the tip vortex and trailing vortex, caused by trailing edge vortices rolling up. Decreasing *f* leads to the higher intensity of vortices, especially, the tip-clearance vortices are stronger in the pump-jet with decelerating duct, while the outlet of pump-jet with accelerating duct shares the stronger rolling up process. The rotor trailing wake flows through the stator blades and becomes more uniform with the decline of intensity of duct-induced vortex, especially pronounced at *f* = 0.25t. Additionally, increasing α enhances the intensity of trailing vortices, whereas the vortex distribution occurs more un-uniform caused by the stronger rotor trailing vortices. The instability of tip vortices becomes intenser in the pump-jet with accelerating duct, even with bifurcation.

Ducts with *f *< 0 or *α *> 0° act as accelerating ducts while those with *f *> 0 or *α *< 0°act as decelerating ducts, except for the duct with camber *f *= 0 or *α *= 0° which is close to a zero-accelerating duct. Within a certain range, increasing *f* or decreasing *α* can both improve performance, but exceeding this range can lead to a decrease in performance. In this study, the pump-jet with *f *= 0.25t performs best for the highest *η* and thrust, as well as the relatively more uniform transient flow field. Generally, decelerating duct exhibits superior propulsive efficiency, lower likelihood of cavitation erosion, and shares the larger fluctuation, with the gentler tip-clearance flow. It performs better at high *J*, but relative more poorly at low *J*. On the other hand, accelerating duct achieves higher outlet velocity and performance more stable at low *J*. However, the instability of tip vortices is intenser, and it experience greater energy loss at high *J*. Therefore, decelerating ducts are more suitable for high-speed underwater vehicles, while accelerating ducts perform better on the low-speed one.

## Data Availability

All data generated or analysed during this study are included in this published article.
